# A novel risk score model based on fourteen chromatin regulators-based genes for predicting overall survival of patients with lower-grade gliomas

**DOI:** 10.3389/fgene.2022.957059

**Published:** 2022-09-26

**Authors:** Yongfeng Zhang, Beibei Yu, Yunze Tian, Pengyu Ren, Boqiang Lyu, Longhui Fu, Huangtao Chen, Jianzhong Li, Shouping Gong

**Affiliations:** ^1^ Department of Neurourgery, The Second Affiliated Hospital of Xi’an Jiao Tong University, Xi’an, China; ^2^ Department of Thoracic Surgery, The Second Affiliated Hospital of Xi’an Jiao Tong University, Xi’an, China

**Keywords:** low grade glioma, chromatin regulators, prognostic signature, tumor immune microenvironment, tumor mutation burden, single cell analysis

## Abstract

**Background:** Low grade gliomas(LGGs) present vexatious management issues for neurosurgeons. Chromatin regulators (CRs) are emerging as a focus of tumor research due to their pivotal role in tumorigenesis and progression. Hence, the goal of the current work was to unveil the function and value of CRs in patients with LGGs.

**Methods:** RNA-Sequencing and corresponding clinical data were extracted from The Cancer Genome Atlas (TCGA) and the Chinese Glioma Genome Atlas (CGGA) database. A single-cell RNA-seq dataset was sourced from the Gene Expression Omnibus (GEO) database. Altogether 870 CRs were retrieved from the published articles in top academic journals. The least absolute shrinkage and selection operator (LASSO) algorithm and Cox regression analysis were applied to construct the prognostic risk model. Patients were then assigned into high- and low-risk groups based on the median risk score. The Kaplan–Meier (K-M) survival curve and receiver operating characteristic curve (ROC) were performed to assess the prognostic value. Sequentially, functional enrichment, tumor immune microenvironment, tumor mutation burden, drug prediction, single cell analysis and so on were analyzed to further explore the value of CR-based signature. Finally, the expression of signature genes were validated by immunohistochemistry (IHC) and quantitative real-time PCR (qRT-PCR).

**Results:** We successfully constructed and validated a 14 CRs-based model for predicting the prognosis of patients with LGGs. Moreover, we also found 14 CRs-based model was an independent prognostic factor. Functional analysis revealed that the differentially expressed genes were mainly enriched in tumor and immune related pathways. Subsequently, our research uncovered that LGGs patients with higher risk scores exhibited a higher TMB and were less likely to be responsive to immunotherapy. Meanwhile, the results of drug analysis offered several potential drug candidates. Furthermore, tSNE plots highlighting the magnitude of expression of the genes of interest in the cells from the scRNA-seq assay. Ultimately, transcription expression of six representative signature genes at the mRNA level was consistent with their protein expression changes.

**Conclusion:** Our findings provided a reliable biomarker for predicting the prognosis, which is expected to offer new insight into LGGs management and would hopefully become a promising target for future research.

## 1 Introduction

Gliomas are the most common primary tumors of the central nervous system. Despite the prognosis of LGGs being more favorable than glioblastoma, nearly 70% of LGGs grow unceasingly and usually transform to higher grades of malignancy (van den Bent 2014). The time to progression can range from a few months to several years, with median survival for LGGs patients varying from 5 to 10 years (Vitucci et al., 2017). In recent years, several classical molecular phenotypes are applied in the stage of LGGs management, such as isocitrate dehydrogenase 1 (IDH1), O-6-methylguanine-DNA methyltransferase (MGMT) and 1p/19q codeletion. There are many therapies targeting these markers designed in clinical trials, whereas the overall survival (OS) of LGGs shares unmet needs for effective treatment. Hence, the identification of novel targets for LGGs management remains imperative.

Epigenetic modification interferes with transcriptional gene signatures, and abnormal patterns are related to tumorigenesis (Lu et al., 2018). In this regard, the function of CRs is of great interest. According to the roles in epigenetics, CRs can be categorized into three main types: histone modifiers, chromatin remodelers, and DNA methylators. Under specific conditions, these three categories may synergize with each other or take precedence (Plass et al., 2013). Previous studies have demonstrated that CRs involved in a wide spectrum of biological phenomena, such as apoptosis (Sui et al., 2021), inflammation (Ding et al., 2019), autophagy (Li et al., 2020), proliferation (Yan et al., 2020), ferroptosis (Zhang et al., 2018), etc. As a promising therapeutic target, CRs were reported to be closely associated with a myriad of diseases, including neurodegenerative diseases (Li et al., 2020) and multiple types of cancers (Au et al., 2012; Chen et al., 2015; Li et al., 2017).

The association between glioma and CRs has been reported (Bao et al., 2017). However, few studies have comprehensively elaborated the correlation between CRs and LGGs. Herein, our current work aimed to construct a CRs-related signature and assess the values in LGGs via bioinformatic analysis.

By identifying differentially expressed-CRs (DE-CRs), we successfully developed and validated a risk model, which has potential prognostic value for LGGs patients. Furthermore, function analysis was performed between high- and low-risk groups to uncover the potential pathway. Subsequently, Given the tumor microenvironment is involved in the pathological process, we identified the relationship between the immune landscape and prognostic signature. Similarly, we quantified the differences in tumor mutation burden (TMB) between the two groups. Moreover, we also screened 10 small molecule drugs, which may be beneficial to the treatment of patients with LGG. Additionally, tSNE plots highlighting the magnitude of expression of the genes of interest in the cells from the scRNA-seq assay. Ultimately, transcription expression of six representative signature genes at the mRNA level was consistent with their protein expression changes.

Taken together, we constructed a 14 CR-based signature, which may be expected to provide a new idea for the management of LGGs.

## 2 Methods

### 2.1 Cell culture and reagents

U87 and U251 cells lines (Chinese Academy of Medical Sciences, Beijing, China) were cultured in DMEM/high glucose supplemented with 10% fetal bovine serum (FBS, Life Technologies, Carlsbad, CA, United States). NHA were purchased from he China Academia Sinica Cell Repository (Shanghai, China) and cultured in RPMI-1640 medium with 10% FBS. All the cells were cultured in a humidified incubator (37°C, 5% CO2).

### 2.2 Dataset and source

The RNA-seq expression data and corresponding clinical data sheets of LGGs were retrieved from the TCGA(https://portal.gdc.cancer.gov/) database as a training cohort. Thereafter, mRNA expression and relevant clinicopathological information of LGGs tissues were downloaded from the CGGA database (http://www.cgga.org.cn) for validation (Table 1). Furthermore, the dataset of somatic simple nucleotide was also obtained from the TCGA database for analysis of TMB. Then, patients without prognostic information were excluded from this analysis. Ultimately, we harvested a TCGA training set with 515 patient samples and a CGGA validation set including 568 patient samples.

**TABLE 1:** Overview of clinical features of LGG data set.

Variables		TCGA (452 samples)		CGGA (499 samples)	
		Cases	Percentage	Cases	Percentage
Age	<41	215	47.6%	264	52.9%
	≥41	237	52.4%	235	47.1
Gender	Female	204	45.1%	209	41.9%
	Male	248	54.9%	290	58.1%
Grade	WHO II	217	48.0%	241	48.3%
	WHO III	235	52.0%	258	51.7%
IDH1 Mutatation	Wildtype	103	22.8%	122	24.4%
	Mutant	349	77.2%	377	75.6%
1p.19q.codeletion	Codeletion	151	33.4%	156	31.3%
	Non-codeletion	301	66.6%	343	68.7%
MGMT promoter	Methylated	375	83.0%	NA	NA
	Unmethylated	77	17.0%	NA	NA
Risk group	Low	226	50.0%	226	45.3%
	High	226	50.0%	273	24.7%
Survival time	(years)	Median	IQR	Median	IQR
		1.67	(1.12–3.06)	3.28	(1.54–6.26)

NA, not applicable; IQR, interquartile range.

### 2.3 Differential analysis

The SVA package was applied to remove batch effects and filter other substandard variations among datasets mentioned above. Genes with |log2FC| > 1 and FDR <0.05 were considered as the significance threshold. Then, the LIMMA package was applied to screen DE-CRs based on R software, the script for the analysis is available in supplementary material (Additional file 12: Supplementary Text S1).

### 2.4 Construction and external validation of a risk-score system

In the context of DE-CRs, univariate Cox regression analysis was performed to identify the prognostic value of CRs, which was subsequently applied in the construction of the risk model by the lasso Cox regression analysis using the “glmnet” R package. Furthermore, we calculated the signature risk score in light of the normalized gene expression levels and constructed a survival risk score model according to the following formula:

$$\text{Risk}\;\text{score}=\sum_{i=1}^{n}\text{expression}\left(i\right)\times \text{coefficient}\left(i\right)$$

Where expression (i), coefficient (i), and n represent the gene expression level, the coefficient of the corresponding mRNA, and the number of genes, respectively. According to the median risk score in the training set, LGGs patients were taken as a cutoff point for dichotomization into low- and high-risk groups. Survival analysis was displayed with R (i.e. survminer packages) by using KM curves. A time-dependent ROC analysis was executed to calculate the AUC and then assess the prognostic ability of the multivariate Cox model. For further validation of the prognostic ability of the model, both ROC and KM curves were also drawn based on a CGGA dataset.

### 2.5 Development and evaluation of the nomogram

To develop a clinically applicable tool for predicting the prognosis of LGGs patients at 1, 3, 5, and 10 years, a nomogram was established according to the results of Cox regression analyses. Finally, five independent prognostic factors, including gender, age, grade, IDH1 status, and risk score were applied to construct a nomogram based on the several classic R packages. Discrimination of the nomogram was assessed with a calibration plot and quantified as a concordance index.

### 2.6 Functional annotation and enrichment analysis

To functionally annotate DE-CRs during the analysis, the GO and KEGG pathway analyses were performed by using R software. Enrichment was considered to have significance with an FDR *p*-value < 0.05. Subsequently, We performed GSEA to detect the underlying molecular mechanisms between the two risk groups mentioned above. Gene sets were permuted 1,000 times for each analysis. Meanwhile, we set the following standards for statistically significant terms: I. FDR<25%; II. *p* value < 0.05.

### 2.7 PPI network analysis

DE-CRs were uploaded to the STRING database to construct the PPI networks. Cytoscape, an open-source software platform, was utilized to generate and visualize complex networks. And the most significant module in the PPI networks was identified using betweeness.

### 2.8 The correlations between CR-based signature and immune cell infiltration

To comprehend the relevance between immune cell infiltration levels and prognostic risk models, four reliable methods were applied to calculate the immune infiltration status of LGGs patients (Chen et al., 2018). Cell-type identification by estimating relative subsets of RNA transcript (CIBERSORT) is a method for quantifying cell fractions from bulk tissue gene expression profiles. In this study, characteristics of infiltrating immune cells between high- and low risk group were analyzed by CIBERSORT and the results are shown in the form of violin diagram. Furthermore, the Wilcoxon test was used to explore the correlation between the expression of the immune checkpoint genes and prognostic risk scores. Eventually, considering the TIMER database is a powerful website that provides versatile analysis of immune cell infiltration, We explored the relevance between fourteen signature genes and immune cells.

### 2.9 TMB analysis

The R package Maftools was used to extract somatic mutational profiles from the TCGA database. At this point, non-synonymous mutation counts were recognized as TMB. Subsequently, mutation number is used to calculate the TMB score for each sample to identify the relationship between the two risk groups. The number of TMBs in each sample was statistically calculated to distinguish high or low TMB group. Next, we compared the differences between the high- and low- TMB groups. Then we stratified patients into four subgroups based on TMB (high or low) and riskscore (low or high). Further we evaluated the synergistic effect of the risk score grouping and the TMB grouping in the prognostic stratification.

### 2.10 Screening for potential small molecule drugs and drug sensitivity analysis

The Drug Signatures database (DSigDB), a new gene set website that relates drugs and their target genes, currently holds thousands of gene sets and consists of unique compounds and corresponding genes (http://tanlab.ucdenver.edu/dsigdb) (Yoo et al., 2015). We uploaded DE-CRs to DSigDB to filter unexcavated small molecule drugs. Besides, to assess the differences in single-drug sensitivity between two risk groups, the GDSC was used to evaluate the drug sensitivity by analyzing the IC50 of drugs on basis of the classical pRRophetic package (Geeleher et al., 2014). A *p*-value of <0.05 was considered to be statistically meaningful.

### 2.11 Characterization of signature genes by single-cell RNA sequencing

The single-cell sequencing data of gliomas were sourced from GSM6094425 and GSM5705583 using the GEO database. Seurat, an R package for single-cell analysis (http://satijalab.org/seurat/), was then used to analyze gene sequencing data. Gene expression in fewer than three cells and in less than 250 cells was ruled out. For quality control, the following criteria were applied for quality control: total UMI count between 2,000 and 6,000, UMI counts>1,000, mitochondrial gene percentage <20% and ribosomal gene percentage >1%. Next, the single-cell sequencing data were dimensionally reduced using principal component analysis (PCA). For clustering and tSNE representations, the first 20 principal components from the scaled data were used. According to DEGs of each cluster by tSNE analysis, by combining automatic and manual annotation based on single R and marker genes reported by previous articles, cells from each cluster were annotated. In order to identify specific cell types, specific cell markers were obtained from the official CellMarker website (http://biocc.hrbmu.edu.cn/CellMarker/). Finally, we characterized the expression of signature genes in different clusters.

### 2.12 Quantitative real-time PCR

Total RNA was isolated from normal human astrocytes (NHA) and U87 cells using TRIzol reagent (Invitrogen, United States). GAPDH was employed as an endogenous control. Total RNA was extracted from each sample using The extracted RNA was reverse transcribed into complementary DNA using PrimeScriptTM RT Master Mix (TaKaRa, Japan). The primer sequences are listed in Table 2. Relative mRNA expression was calculated using the 2-ΔΔCt method and compared to that of the control group (GAPDH mRNA 128 expression). Statistical comparisons were performed using the student’s t-test; differences with *p* < 129 0.05 were considered to be statistically significant.

**TABLE 2:** Specific primers used for quantitative real-time PCR.

Primer	Sequence
GAPDH-F	GGA​GCG​AGA​TCC​CTC​CAA​AAT
GAPDH-R	GGC​TGT​TGT​CAT​ACT​TCT​CAT​GG
TRIM24-F	GAA​GTG​GCT​GGA​CTC​TCT​AAA​C
TRIM24-R	TGC​CGT​AAC​CGG​TAT​GTA​ATC
IDH1-F	GAC​TTG​GCT​GCT​TGC​ATT​AAA
IDH1-R	GGC​CTG​AGC​TAG​TTT​GAT​CTT
LBR-F	CTG​GCA​GTG​AGA​ACC​TTT​GA
LBR-R	AGC​AAC​AGG​AAG​AGG​AAC​AC
HMG20B-F	GGA​GAA​GAA​GAT​CAA​GAA​AGA​AGA​C
HMG20B-R	CTG​GGC​CAG​GAT​TTC​CTT​GAT
USP49-F	TGG​TCT​GGC​CGT​AAT​CAT​CG
USP49-R	CCT​GCA​GCA​GTA​AGG​TTC​CA
RCC1-F	GCT​CCT​TCC​GGG​ACA​ATA​AC
RCC1-R	ACC​TTT​ACC​ACA​GGC​ACA​TC

### 2.13 Validation of signature genes at the protein level

Human Protein Atlas(HPA) database contains transcriptome and proteome sequence data derived from RNA-sequencing analysis and immunohistochemistry analysis, reflecting its important value in protein expression analysis. Six up-regulated genes (TRIM24, IDH1, LBR, HMG20B, USP49, and RCC1) were extracted from CRs-related signature. Following the age and gender matching rules, we tried to validate the protein expression of signature genes between normal and tumor tissues in the HPA database. Additionly, Image analysis was carried out using the free and public domain software ImageJ (NIH Image, Bethesda, MD, United States). Unpaired Student’s t test was used to calculate the *p* values for comparisons of quantitative evaluation of immunohistochemical staining between normal and tumor tissues. All data were analyzed and plotted using GraphPad Prism (GraphPad Software).

### 2.14 Statistical analysis

All statistical analyses were carried out with the RStudio and its appropriate packages. For continuous numerical variables consistent with normal distribution, t-test was used for comparison between two groups, one-way ANOVA was used for comparison of three or more groups, and pairwise comparison was performed after the event. LSD test was used for homogeneity of variance, and Dunnett’s T3 test was used for uneven variance. For continuous numerical variables that did not fit to normal distribution, the nonparametric test method is used for grade data; Mann-Whitney tests is used to comparison between two groups, and the statistics are expressed by Z value; Kruskal–Wallis Test was used for comparison between groups. Categorical variables were expressed as a percentage, applying chi-square test to analyze the differences between groups. And a two-tailed *p* value of <0.05 was considered statistical significance.

## 3 Results

### 3.1 Establishment and external verification of CRs-related prognosis model

**FIGURE 1:**
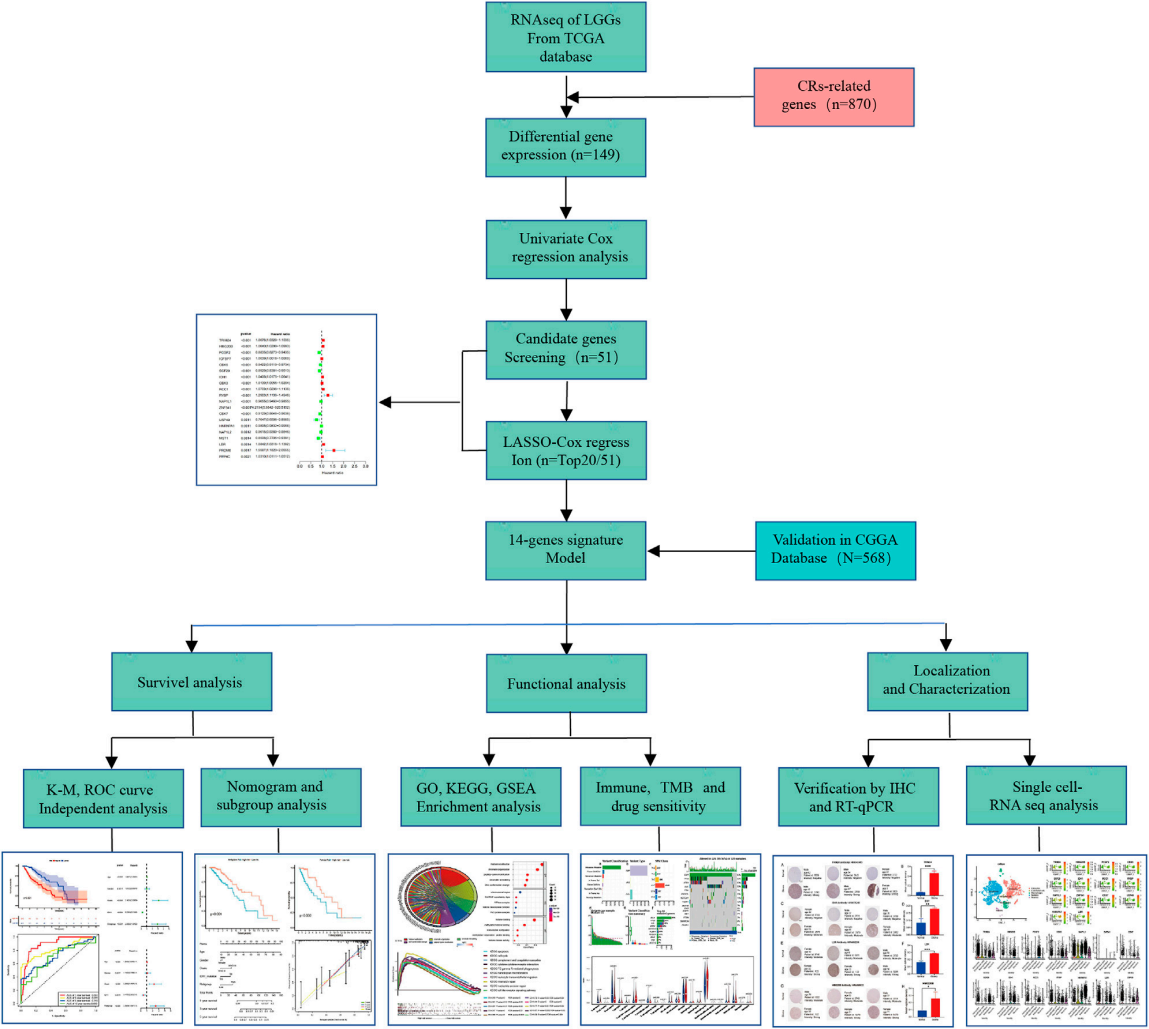
The flowchart of data preparation and analysis.

RiskScore features had the greatest impact on survival rate prediction, which indicated that the risk model based on 14 genes could better predict outcomes. To systematically expound our work, a work flow was summarized in detail (Figure 1). Compared with normal brain tissue, 149 CRs in the TCGA database were identified as DE-CRs, of which 108 were up-regulated and 41 were down-regulated (Supplementary Sheet S1 Part 1). Then, the univariate Cox analysis was applied to screen DE-CRs based on their prognostic value. The results suggested that 51 CRs met the criteria(Supplementary Sheet S1 Part 2–3), top20 genes of 51 CRs were presented in Supplementary Figure S1. Furthermore, based on these filtered genes, we used LASSO-regularized Cox regression to establish a risk model. Finally, 14 DE-CRs (IDH1, TRIM24, HMG20B, PCGF2, CBX6, SGF29, RCC1, RYBP, NAP1L1, ZNF541, CBX7, USP49, HNRNPA1, and LBR), have been utilized to successfully construct prognostic models (Supplementary Table S1). The risk score was calculated by the corresponding coefficient of 14 CRs with the following formula: Risk score = $\sum_{i=1}^{14}expression\left(i\right)\times coefficient\left(i\right)$ .

**FIGURE 2:**
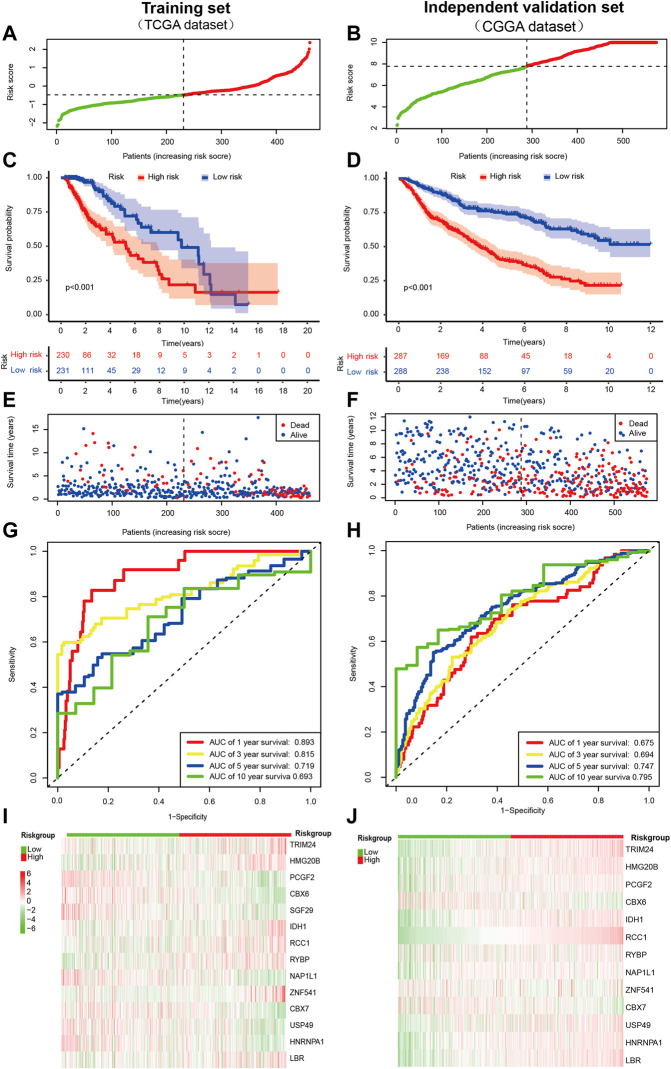
Prognostic value of the CRs-based risk signature in LGG. **(A,B)**Distribution of patients with different risk scores in the training set and external independent validation set. The red and green points represent patients in the high- and low-risk grouop respectively. **(C,D)** Survival analysis of patients in the training set and external independent validation set. The number of patients gradually decreased in both groups as time progressed. The survival curve shows that patients in the high risk group had a poorer outcome than low risk group. **(E,F)** Survival status of patients with different risk scores in the training set and external independent validation set. The red and blue points represent patients who were dead and alive, respectively. As the risk score increased, the survival time of patients decreased and the number of deaths increased. The higher the risk score, the closer the corresponding point is to the right of the horizontal axis and the lower of the vertical axis. **(G,H)** The AUC of time-dependent ROC curves identified the prognostic performance of the risk score. The ROC curve describes the trade-off between sensitivity and specificity of a classifier, with the ideal ROC curve reaching the top left corner. The gray diagonal line in each ROC figure is the baseline, which presents the ROC curve of a random classification. **(I,J)** Distribution of specific gene expression based on risk score. Heatmap of the prognostic signature scores in the training set and external validation set. The red and green parts represent patients in the high and low risk group, respectively.

After that, the patients were divided into a low- (n = 231) and high-risk group (n = 230) based on the median value of risk score in the TCGA cohort(Figure 2A). Our data also demonstrated that the mortality of the high-risk group was significantly higher than the low-risk group (Figures 2C,E), which suggested that a high-risk score was associated with poor OS in the TCGA cohort. As the risk score increased, the survival time of patients decreased and the number of deaths increased. Next, the model’s reliability has been further testified through the ROC curve, and the AUC was 0.893 at 1 year, 0.815 at 3 years, 0.719 at 5 years, and 0.693 at ten yesrs (Figure 2G). ROC curve was used to evaluate the predictive efficacy of the predictive model for patient prognosis. The closer the ROC curve is to the upper left corner, the closer the area is to 1, and the better the classification effect. A diagonal indicates a random guessing method. Finally, we present the heatmap for signature genes between the high- and low-risk group in the TCGA cancer set (Figure 2I).

Furthermore, we also validated the reliability of the CRs-related signature according to the data from the CGGA database preliminarily. Notably, SGF29 was not found in the CGGA database, so it was validated with the remaining signature genes. Likewise, the patients were dichotomized as a low- (n = 288) and high-risk group (n = 287) based on the median value of risk score in the CGGA cohort(Figure 2B). By and large, the results of survival analysis were grossly in line with the training set (Figures 2D,F). Besides, the AUC of time-dependent ROC indicated that CRs-based signature had a better predictive ability (AUC = 0.675, 0.694, 0.747, and 0.795), at one, three, five and 10 years, respectively (Figure 2H). Similarly, we also observed a different gene signature between low-risk and high-risk individuals in CGGA databse. Of note, It was revealed that the expression of most genes was generally consistent with results of TCGA cancer set, such as TRIM24, HMG20B, CBX6, IDH1, RCC1, RYBP, CBX7, and LBR (Figure 2J).

### 3.2 Relationship between CRs-related signature and the clinical features

**FIGURE 3:**
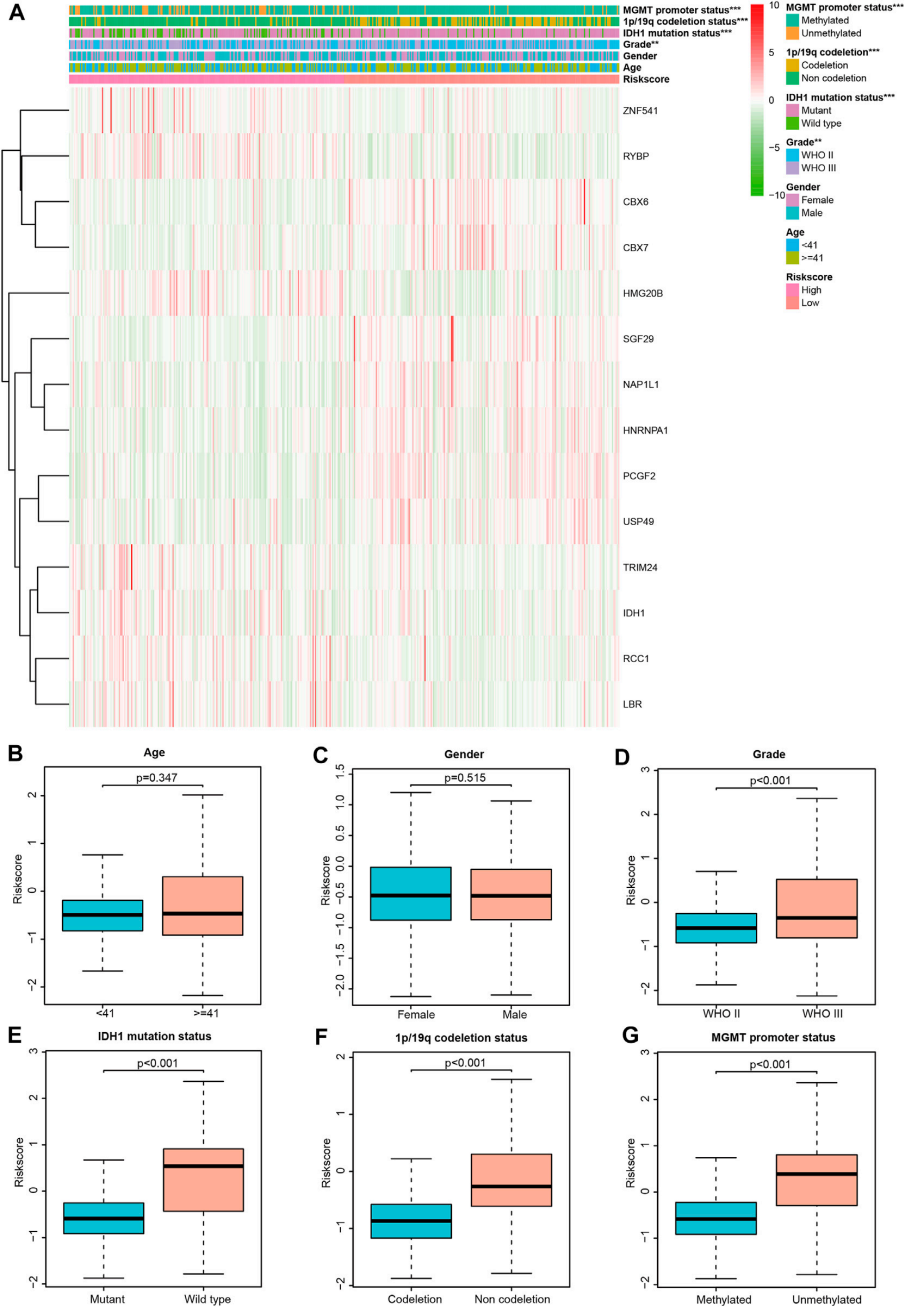
Correlation between CRs-based signature risk scores and clinical-pathological characteristics. **(A)** Heat map of the clinical-pathological characteristics and signature genes expression; **(B–G)** distribution of the risk scores in different cohorts stratified by the clinical-pathological characteristics.

**FIGURE 4:**
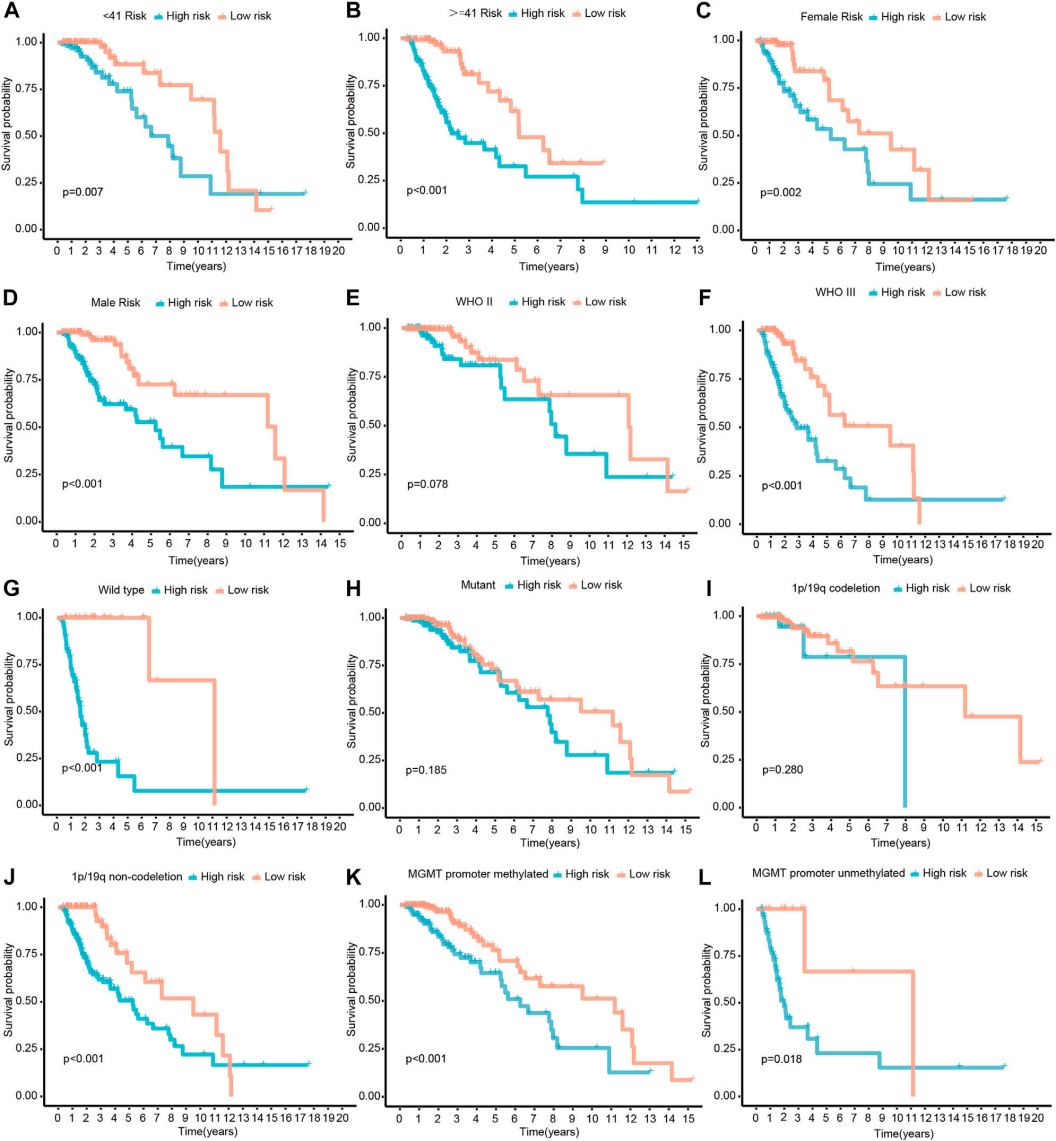
Survival differences stratified by Age, Gender, Grade, IDH1, 1p/19q codeletion status or MGMT promoter status between the high- and low-risk groups. **(A,B)** The patients were divided into < 41- and > = 41-years subgroups according to their age. **(C,D)** The patients were divided into female and male subgroups according to their gender. **(E,F)** The patients were divided into WHO II and III subgroups according to WHO classification. **(G,H)** The patients were divided into mutant and wild type subgroups according their IDH1 mutatation status. **(I,J)** The patients were divided into 1p/19q codeletion and non-codeletion subgroups according to their 1p/19q codeletion status. **(K,L)** The patients were divided into MGMT promoter methylated and unmethylated subgroups according to their MGMT promoter methylation status.

To determine whether CRs-related signature affects the clinical characteristics of LGGs, we used the Chi-square test to compare differences between the two risk groups. The result suggested that there was a significant difference in IDH1 mutation status, 1p/19q codeletion status, MGMT promoter and World Health Organization (WHO) grade, whereas gender and age do not (Figures 3A,B). Furthermore, each grid in the heat map represents the expression level of each signature gene corresponding to different clinical factors and risk groups. The greener the color of the heatmap, the lower the gene expression, and the redder the color of the heatmap, the higher the gene expression. Sequentially, stratified analysis was performed to explore the prognostic difference of the CRs-related signature in subgroups. The patients were divided into two subgroups according to clinicopathological factors of the patients. Following that, patients were dichotomized according to their median risk scores into low- or high-risk subgroups, respectively. The results showed that CRs-related signature have higher practical value in predicting prognosis under different clinical characteristics. The figure shows that the expression of <41-, > = 41-years, female, male, WHO grade III, wild type, 1p/19q non-codeletion, MGMT promoter methylated and unmethylated subgroups are significantly different between high and low-risk groups (Figure 4).

### 3.3 Independent prognostic factors of OS

**FIGURE 5:**
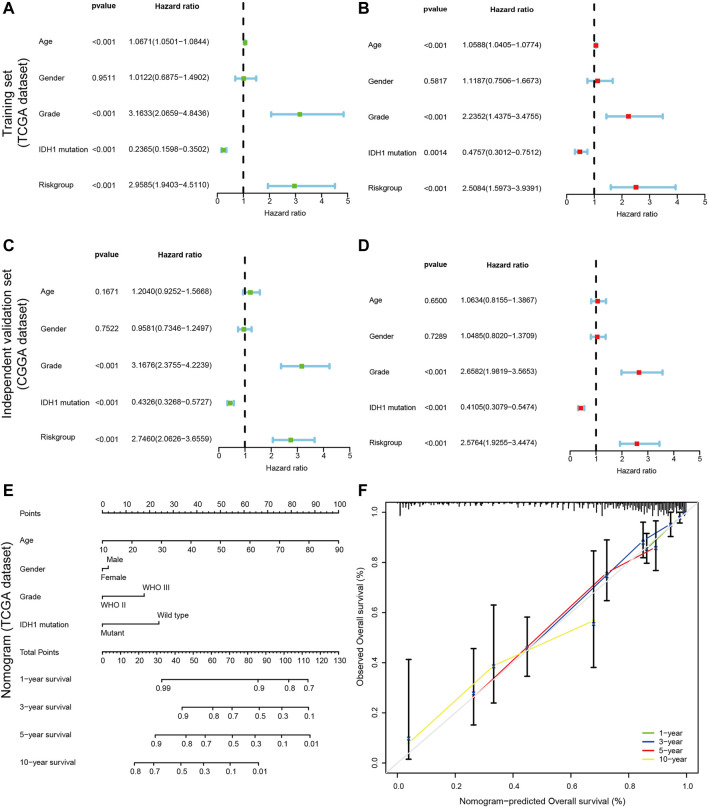
Construction of a nomogram for predicting the Personalized OS of patients with LGG. **(A,C)** Univariate Cox regression analysis evaluating the independent prognostic value of the risk score and clinicopathological in terms of OS in the training set (TCGA cohort) and external independent validation set(CGGA cohort); **(B,D)** Multivariate Cox regression analysis assessing the independent prognostic value of the risk score and clinicopathological in terms of OS in the training set and external independent validation set; Solid squares represent the Hazard Ratios (HR) of death, and horizontal lines represent the 95% confidence intervals(CIs). All *p* values were calculated using Cox regression hazards analysis. Note that if the 95% confidence interval (CI) crosses the vertical dotted line, the factor is not statistically significant. **(E)** The nomogram was applied to predict the OS of patients with LGG at 1, 3, 5 and 10 years. The value of each of variable was given a score on the point scale axis. A total score could be easily calculated by adding each single score and, projecting the total score to the lower total point scale, we were able to estimate the probability of patients with LGG; **(F)** The calibration curves for the nomogram to predict 1-, 3-, 5- and 10-years OS. The x-axis represents the nomogram-predicted probability and y-axis represents the actual probability of LGG patients. Ideal prediction would correspond to the 45° gray dashed line. The solid line represents the entire cohort (n = 452), vertical bars indicate 95% confidence intervals (CIs), and the oppositely placed triangle symbols indicate bias-corrected estimates, indicating observed nomogram performance. The calibration plots presented good agreement between the nomogram prediction and actual observation.

In the current analysis, we performed both univariate and multivariate Cox analyses to determine whether CRs-based signature can be used as an independent prognosis factor. For univariate cox regression analysis, A *p*-value less than 0.05 (p < 0.05) signified that the variable is a prognostic factor for overall survival statistically. Similarly, as for multivariate Cox regression analysis, A *p*-value less than 0.05 (p < 0.05) suggested that the variable is as independent prognostic factor for overall survival. The univariate and multivariate Cox regression revealed that WHO grade, IDH mutation status, and risk score were significantly correlated with poor OS both in the training set and external independent validation set, older age was associated with shorter overall survival times in TCGA dataset but not in CGGA dataset (Figures 5A–D). So, in other words, the risk score based on hub genes can be seen as an independent prognostic marker. Likewise, tumor grade, and IDH mutation status were also remarkably related to prognosis. All of these mentioned above showed that CR-based signature can be used as independent prognostic factors of LGGs.

### 3.4 Establishment of a nomogram

In the process of quantifying individual risk in a clinical setting by combining multiple risk factors, the nomogram acts as a powerful tool for assessment. By synthesizing 14 CRs-related signature, a nomogram was constructed based on risk score, age, tumor grade, and IDH1 mutation status to predict the probability of 1-, 3 -, 5, and 10-years overall survival rates (Figure 5E). The calibration curve showed that the actual survival time of patients was broadly consistent with model predictions. Meanwhile, the calculated concordance index was to be 0.833 (95% CI = 0.790–0.876), which suggested the good predictive power of the nomogram (Figure 5F).

### 3.5 GO and KEGG pathway enrichment analyses

**FIGURE 6:**
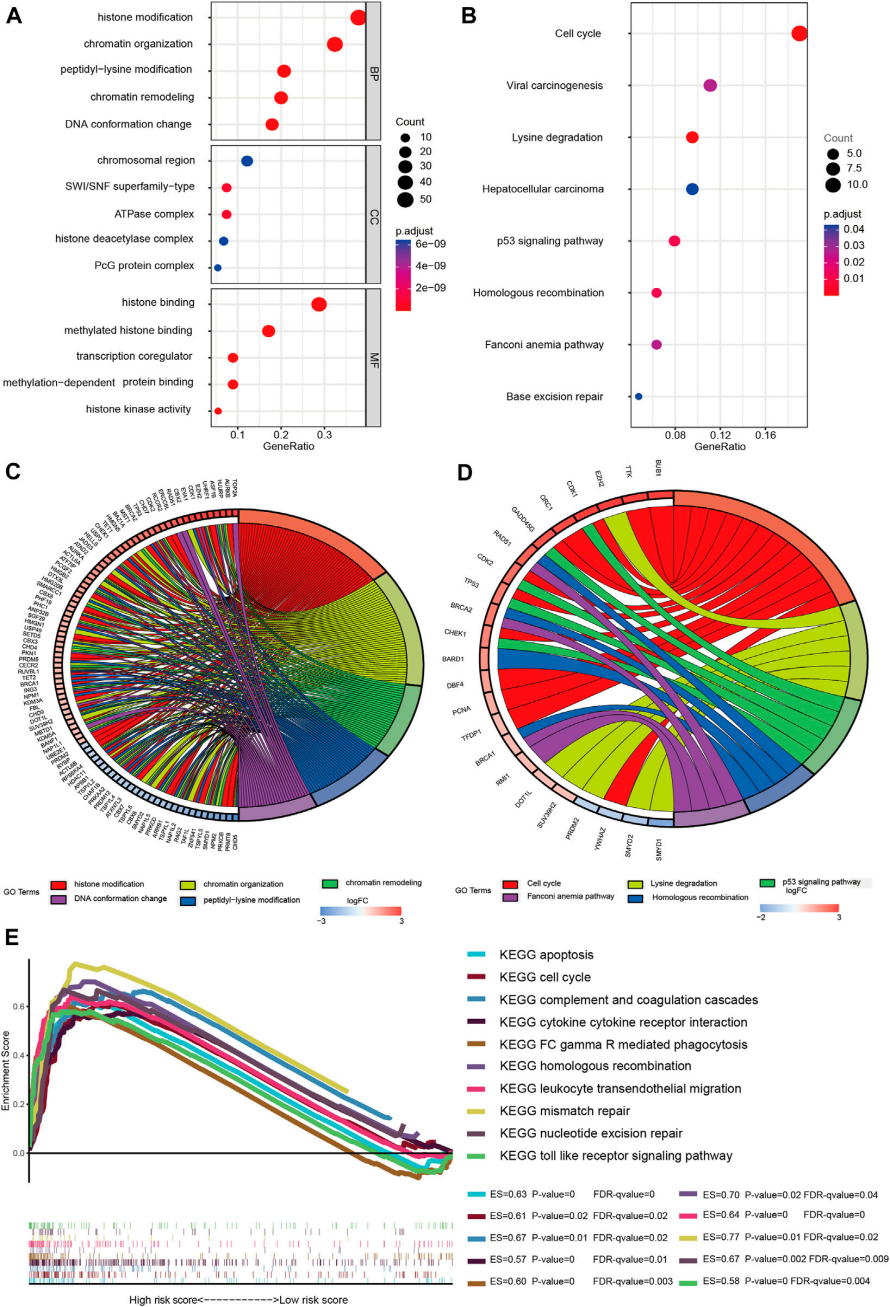
Gene enrichment analysis. **(A)** Enriched GO functions of DEGs. GO: Gene Ontology; BP: biological process; CC: cellular component; MF: molecular function; **(B)** KEGG-enriched analysis; The bubble size represents the number of the target in the enriched pathway terms, bubble color represents the pathway’s *p* value; **(C)** Chord plot of biological process; **(D)** Chord plot of KEGG pathways; Name of identified genes out of the DEGs associated with each pathway are shown. **(E)** GSEA analysis was performed in the high-risk group (nominal *p* value < 0.05, FDR < 0.25). ES, enrichment score; FDR, false discovery rate.

Both GO and KEGG analyses were performed for functional annotation of DE-CRs. The results of BP(Biological Process) analysis indicated that differential genes are enriched in histone modification, chromatin organization, peptidyl-lysine modification, chromatin remodeling, and DNA conformation change. CC(Cellular Component) analysis showed that these CRs were intensively involved in the chromosomal region, SWI/SNF superfamily-type complex, ATPase complex, histone deacetylase complex, and PcG protein complex. The feature DE-CRs were also presented in MF(Molecular Function), such as histone binding, methylated histone binding, transcription coregulator activity, etc (Figures 6A,C). Moreover, the KEGG analysis revealed that the genes were mainly located in the Cell cycle, Viral carcinogenesis, Lysine degradation, Hepatocellular carcinoma, and p53 signaling pathway (Figures 6B,D). Meanwhile, The gene names were mapped to the pathway based on information provided by GO and KEGG by the chord plot (Figures 6C,D).

### 3.6 GSEA

To clarify the potential molecular mechanisms of the signature genes, GSEA analysis was performed both in the two risk groups. The results suggested that several immune-oncologic and metabolism related pathways, such as complement and coagulation cascades, cytokine cytokine receptor interaction, FC gamma R mediated phagocytosis, leukocyte transendothelial migration, and toll like receptor signaling pathway were enriched in the high-risk group. In parallel, some pathways that related to cell cycle regulation, proliferation and apoptosis were also enriched in the high-risk group, such as apoptosis, cell cycle, homologous recombination, mismatch repair and nucleotide excision repair signaling pathway (Figure 6E). Also, the GSEA results of the low-risk group are shown in the supplementary material, the enrichment score (ES), nominal enrichment score (NES), nominal *p*-value (NOM p-val), and false discovery rate (FDR) for each gene set are shown (Supplementary Sheet S2). These findings have revealed the potential role of CRs-related genes in the carcinogenesis, tumor microenvironment, and metabolic response of LGGs.

### 3.7 PPI network construction

Then we constructed a PPI network to further explain the potential interaction among DE-CRs and obtained 87 hub genes with the most interaction (Supplementary Figure S2). Size and location of the circle represent the importance of genes, the larger circle area, the closer to the central of the disk center, the more important the gene is.

### 3.8 Immune cell infiltration and immune checkpoint analysis

**FIGURE 7:**
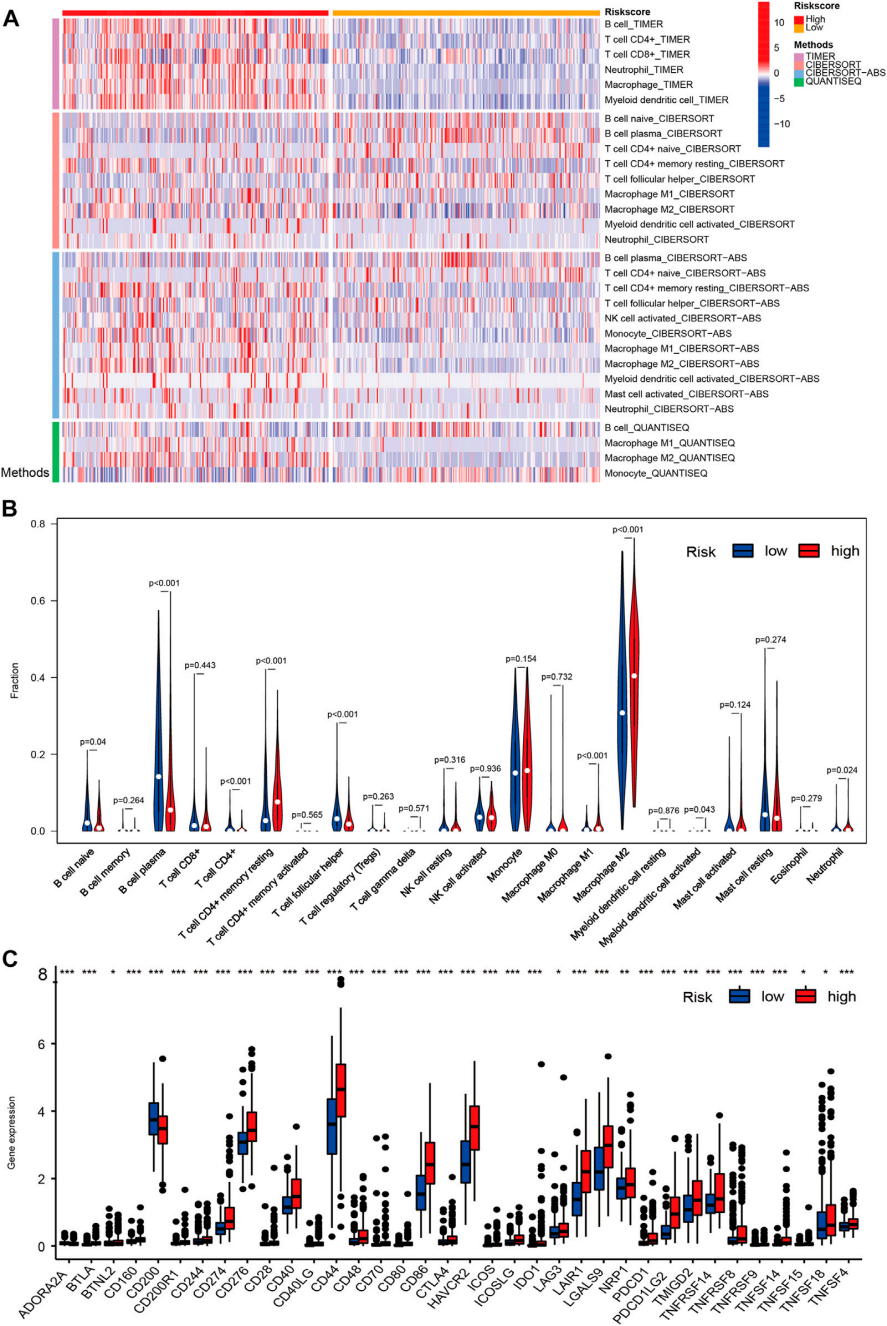
The overview of immune infiltration and expression of immune checkpoints in LGG patients with different risk scores. **(A)** Heat map showing differences in the infiltration of immune cell types calculated via four different algorithms in the low- and high-risk groups. On the left edge of the heat map, the color bars from top to bottom represent the four algorithms used in the calculation. As shown in the illustration on the right, plum, orange, sky blue and green represent TIMER, CIBERSORT, CIBERSORT-ABS and QUANTISEQ, respectively. On the top edge of the heat map, the color bars from red (left) to yellow (right) represent the high- and low risk group. **(B)** Violin plots showing infiltration fractions of different immune cells in the high- and low-risk groups by CIBERSORT. **(C)** Violin plots showing the expression level of immunocheckpoints in high- and low-risk groups, (∗0.01 < *p* < 0.05, ∗∗0.001 < *p* < 0.01, and ∗∗∗*p* < 0.001).

To further explore the efficiency of signature genes on the status of the tumor microenvironment, four kinds of algorithms were applied between two risk groups (Figure 7A). Furthermore, the result of CIBERSORT suggested that the proportions of B cell naive, T cell CD4^+^, T cell CD4^+^ memory resting, T cell follicular helper were higher in the low-risk group, while the B cell plasma, Macrophage M1, Macrophage M2, Myeloid dendritic cell activated and neutrophil had higher proportions in the high-risk groups (Figure 7B). We then investigated the expression levels of immune checkpoints among samples with different risk groups, the outcomes suggested a prominent difference in the expression of CTLA4, PDCD1LG2, PDCD1, TMIGD2, and CD274, etc (Figure 7C). The results unveiled that CRs-related genes might be potential indicators for the regulation of immune activity in the tumor immune microenvironment.

### 3.9 TIMER analysis

TIMER database was performed to identify the correlation between 14 prognostic CRs and several kinds of immune cells. The results suggested that TRIM24, IDH1, RCC1, and LBR were positively related to infiltrating immune subsets, such as CD4^+^ T cells, B cells, dendritic cells, and CD8^+^ T cells. Likewise, PCGF2, SGF29, CBX6, NAP1L1, CBX7, USP49, and HNRNPA1 were negatively related to macrophage, dendritic cells, B cells, neutrophils, and CD4^+^ T cells (Supplementary Figure S3).

### 3.10 Differential tumor mutation burden in different risk groups

**FIGURE 8:**
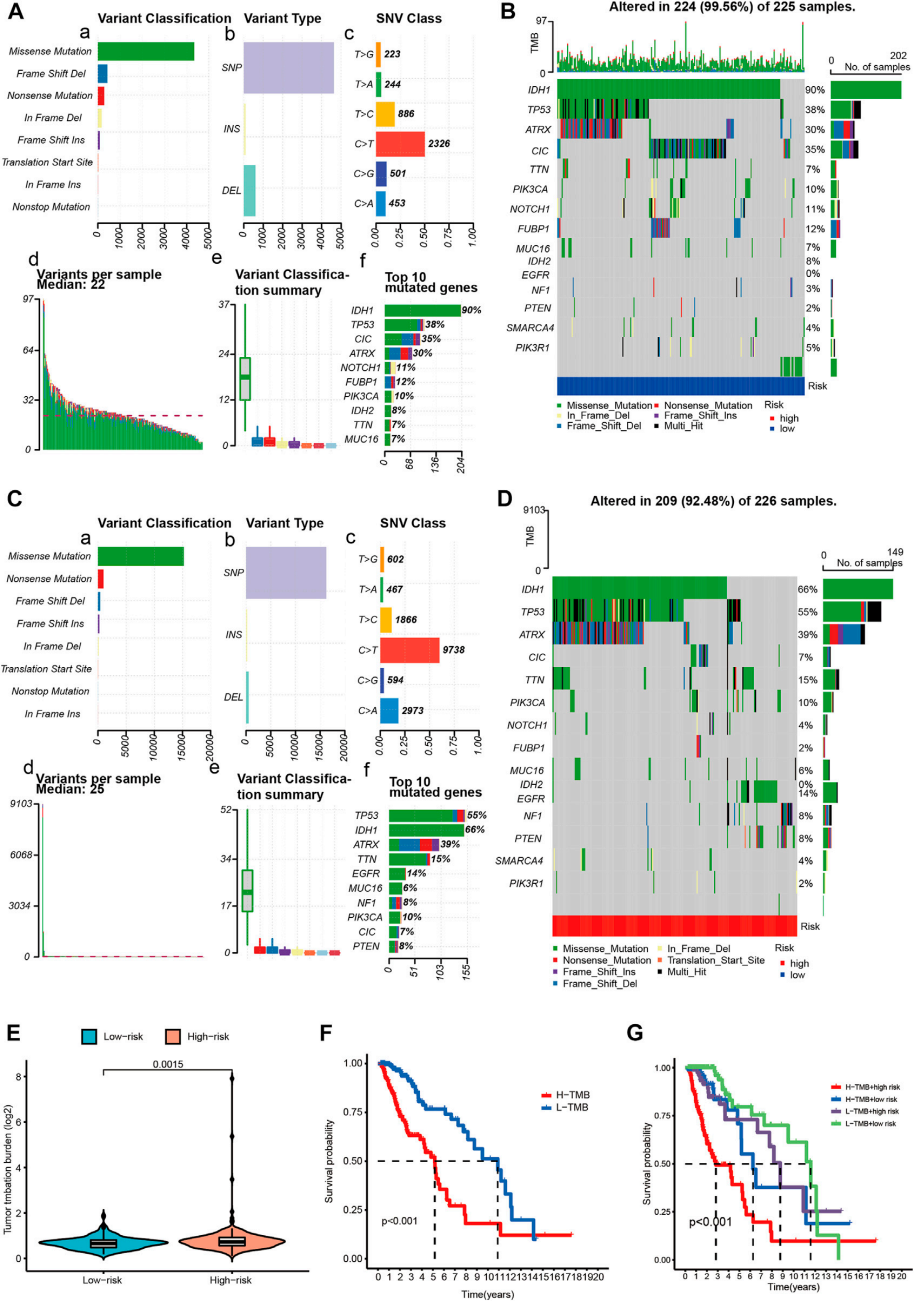
Overview of LGG single nucleotide variant information. **(A,C)** Classification of mutation types according to different TMB in specific samples between low **(A)** and high **(C)** risk score groups. **(B,D)** OncoPrint of distinctly mutated genes in low **(B)** and high **(D)** risk subgroups; **(E)** TMB difference between low- and high-risk subgroups. **(F)** Survival of patients with LGG based on the high- and low-TMB. **(G)** Survival of patients with LGG based on the TMB and risk scores. TMB-H, high tumor mutation burden; TMB-L, low tumor mutation burden.

The WHO classification of CNS tumors includes somatic mutation and copy number variation (CNVs), combined with histological features to make a summary diagnosis. A high TMB is related to better outcome of immunotherapy. In the current analysis, we analyzed the somatic mutations and identified the differences between the two risk groups. As shown in Figures 8A,C, missense mutation was the most common type of variant classification (Figure 8A,C-a), and single nucleotide polymorphisms(SNP) occurred more frequently than insertions(INS) or deletions(DEL) (Figure 8A,C-b). Moreover, C > T was the most common single-nucleotide variation (SNV) (Figure 8A,C-c). Then we counted the number of base changes in each sample, with different colors representing different types of mutations. According to the value of TMB, we divided the LGG patients into the high and low TMB groups. In all, the TMB in the high risk group was higher than that in the low risk group (Figure 8E). Consistently, missense mutation(green) was the most common type of variant classification on average in each sample (Figure 8A,C-e). And then, the results revealed that the low-risk group had higher mutation rates (Altered in 224 (99.56%) of 225 samples) than the high-risk group (Altered in 209 (92.48%) of 226 samples) (Figures 8B,D). Moreover, some important differentially mutated genes with different risk scores were compared (Figure 8A,C-f), TP53, ATRX, PTEN, and EGFR with more significant mutations in the high-risk group whereas NOTCH1, CIC, IDH1, and so on with more remarkable mutations in the low-risk group. Of note, IDH1 not only was the gene with the highest percentage of mutated genes but also seen among the 14-CRs based signature. Additionally, K-M survival analysis demonstrated significant differences in survival when the patients were stratified into high TMB vs. low TMB groups. And the results suggested that patients with lower mutation load may gain better prognosis (Figure 8F). Also, the low-TMB and low-risk groups had the highest overall survival rates, while the high-TMB and high-risk groups had the lowest (Figure 8G).

### 3.11 Explore potential small molecule drugs

Based on the DSigDB database, we found the 10 most potential small molecule drugs closely related to CRs-related signature. They were cephaeline HL60 DOWN, emetine HL60 DOWN, cephaeline MCF7 DOWN, piroxicam CTD 00006571, emetine MCF7 DOWN, formaldehyde CTD 00006001, NSC95682, phenobarbital CTD 00006510, hydrogen peroxide CTD 00006118 and piperlongumine HL60 UP, etc (Supplementary Table S2).

### 3.12 Drug sensitivity analysis

**FIGURE 9:**
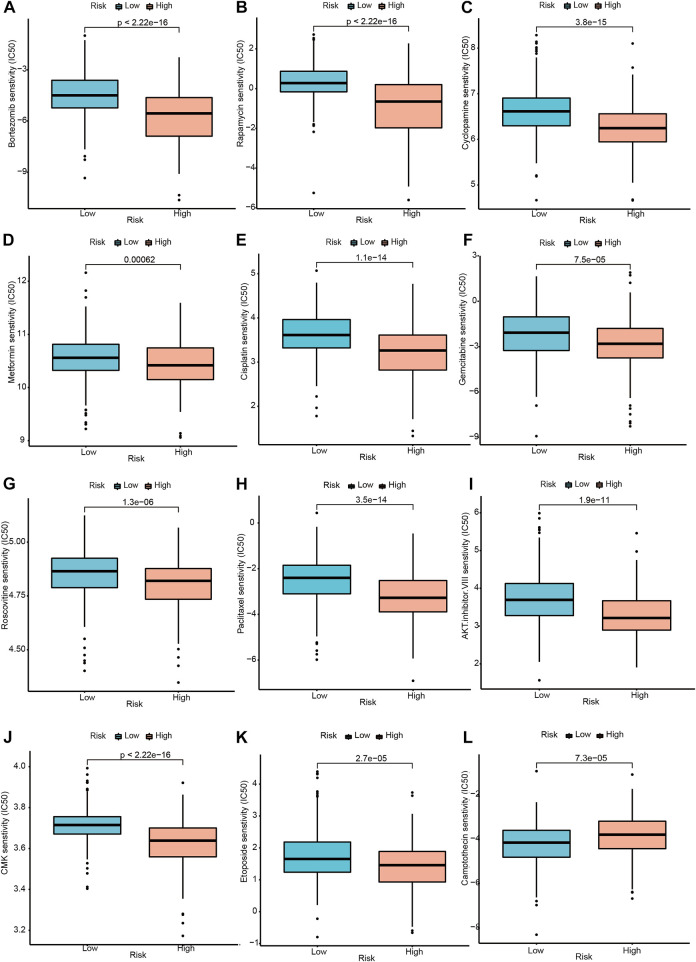
Drug sensitivity analysis between the high and low-risk groups. **(A–K)** Drugs with higher drug sensitivity in the low-risk group are shown. **(L)** Drugs with higher drug sensitivity in the high-risk group are shown.

Moreover, according to the risk score model based on the signature genes, high risk scores correlate with lower IC50 values for Bortezomib, Rapamycin, Cyclopamine, Metformin, Cisplatin, Gemcitabine, Roscovitine, Paclitaxel, AKT. inhibitor.VIII, CMK, and Etoposide, whereas they were related to a higher IC50 for Camptothecin. The IC50 represents the concentration of an inhibitor required to inhibit cancer cells by 50 percent. The lower IC50, the better drug sensitivity. Patients with higher risk scores were more sensitive to Bortezomib, Rapamycin, Cyclopamine, Metformin, Cisplatin, Gemcitabine, Roscovitine, Paclitaxel, AKT. inhibitor.VIII, CMK, and Etoposide, and so on. Similarly, Patients with lower risk scores were more sensitive to Camptothecin. (Figure 9 p < 0.001).

### 3.13 Overview of the scRNA-Seq data generated from gliomas

**FIGURE 10:**
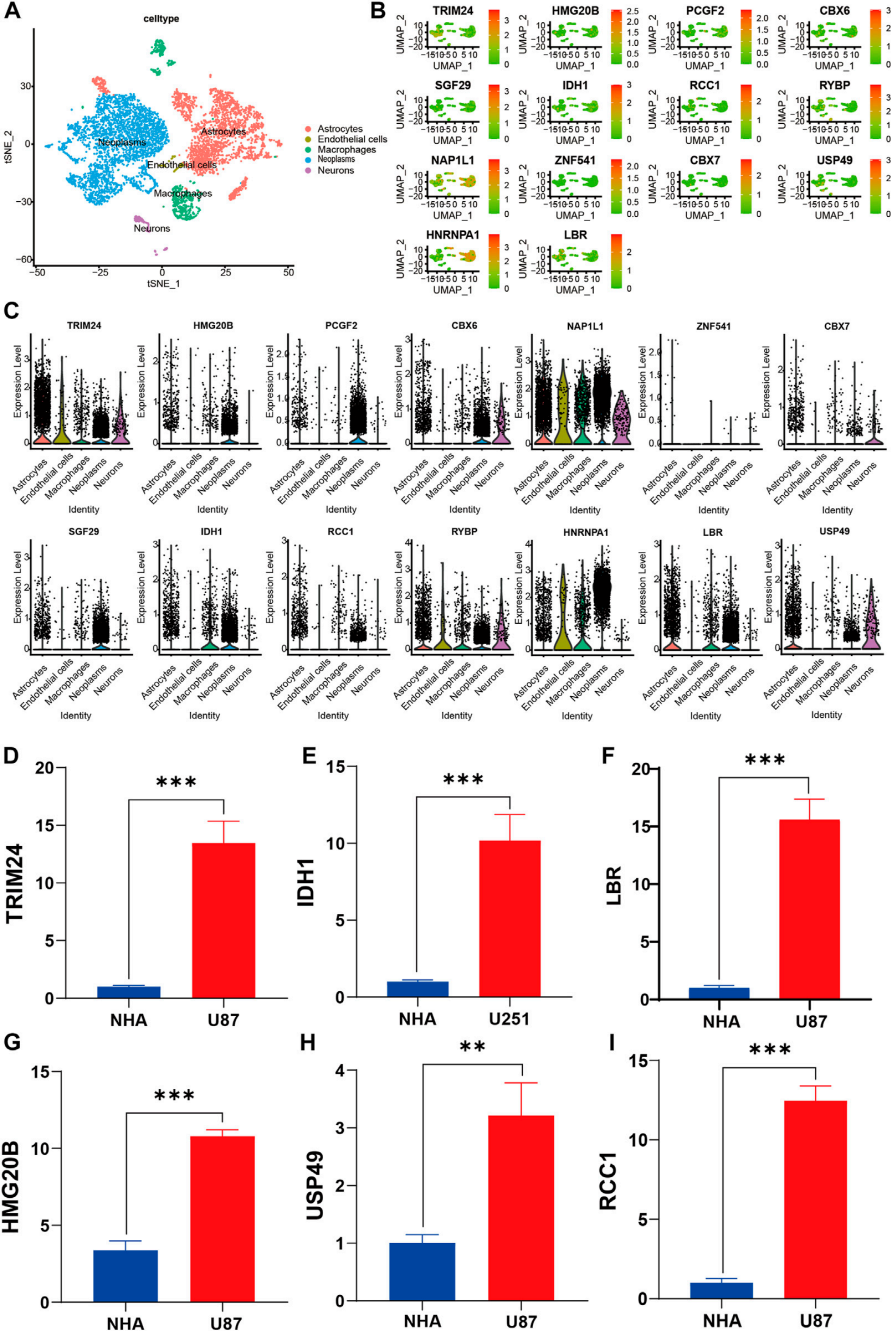
Verification and characterization by sc-RNA seq and RT-qPCR. **(A)** tSNE plots of cells generated from gliomas. The plots are colored by cell cluster, and the cells are clustered into five sub-clusters. Each dot represents a single cell. **(B)** The expression of signature genes in gliomas visualized in tSNE. **(C)** Violin plots depicting the expression of signature genes in clusters of gliomas. The y axis shows the normalized read count. t-SNE: t-distributed stochastic neighbor embedding. **(D–I)** RT-qPCR data demonstrated that six representative signature genes was significantly upregulated in glioma cell lines compared with NHA cell lines. **p < 0.01; ***p < 0.001; NHA, normal human astrocyte; U87 and U251, human glioma cell line.

The single-cell data were pre-filtered using the Seurat package, yielding a total of 5,977 and 5,082 cells from GSM6094425 and GSM5705538, respectively. Following a second filtration process conducted with UMI counts, mitochondrial content, and ribosomal gene content, there were 4,364 and 3,730 cells that remained from GSM6094425 and GSM5705538 (Supplementary Figure S4A,B). After that, the top 20 hypervariable genes were labeled (Supplementary Figure S4C). By using principal component analysis (PCA) to reduce dimensionality, we kept the top 20 components for further tSNE and UMAP dimension reduction, fifteen cell subpopulations were obtained (Supplementary Figure S4D, Supplementary Figure S5A). The percentages of different cell subpopulation in total cells are indicated on the ratio diagram (Supplementary Figure S5C). Seurat was used to identify the DEGs within each cluster, and the top 5 genes in each cluster were visualized with a heatmap and Bubble chart (Supplementary Figure S5D,E). We manually annotated above clusters as the following five cell types: 1) Neoplasm (SOX2, TPI1, PARP1, CCND2, and SMOC1); 2) Neurons (MAP2, STMN2, and GAD2); 3) Macrophages (CD74, CD86). Astrocytes and endothelial cells were annotated using the SingleR algorithm (Figure 10A, Supplementary Figure S5B). To investigate the expression of signature genes in distinct cells, we visualized these with tSNE and violin plots (Figures 10B,C). Most of siganture genes are highly expressed in tumor cells, such as HMG20B, PCGF2, CBX6, SGF29, IDH1, TRIM24, LBR, and HNRNPA1, which is basically consistent with the results at the RNA and protein level.

### 3.14 Verification of the six representative signature genes using RT-qPCR and immunohistochemistry assay

**FIGURE 11:**
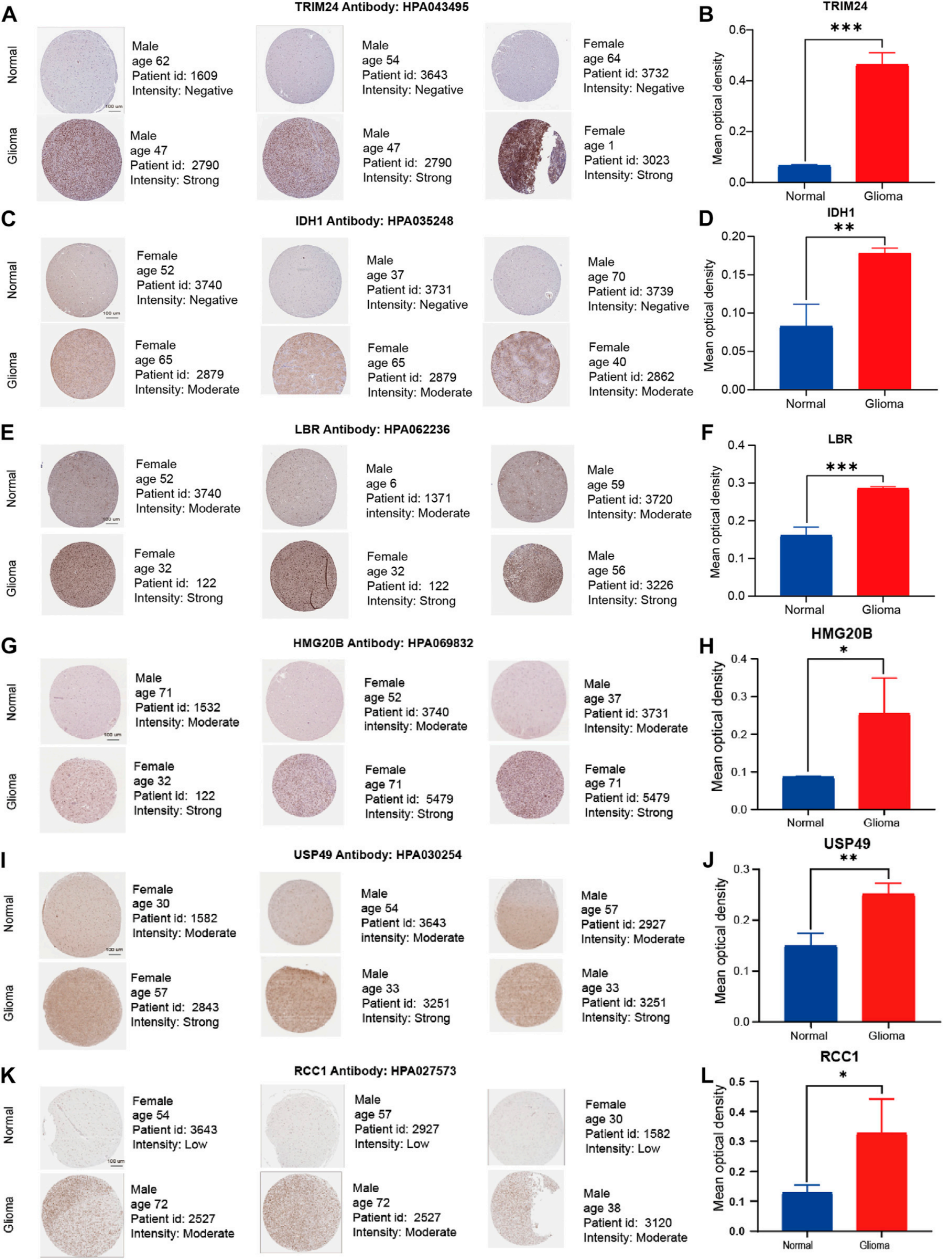
Representative immunohistochemistry images of TRIM24, IDH1, LBR, HMG20B, USP49, and RCC1 in the normal cerebral cortex and glioma tissue derived from the HPA database. **(A–L)** The expression quantity of representative genes in the tumor tissues higher than that in the paired normal tissues. Scale bar = 100 μm ∗*p* < 0.05, ∗∗*p* < 0.01, and ∗∗∗*p* < 0.001, compared with the control groups.

We validated expression of six of signature genes at the transcript and protein level. The RT-qPCR assay showed that the six signature genes (TRIM24, IDH1, LBR, HMG20B, USP49, and RCC1) were up-regulated in glioma cell lines in mRNA expression level (Figures 10D–I). It is worth pointing out that compared with NHA cell line, IDH1 is highly expressed in both U87 and U251 Cell Lines, but the expression difference in U87 is more significant than that in U251 Cell line. Therefore, we show the results of U251 compared with normal astrocytes (Figure 10E). Furthermore, the immunohistochemistry assay revealed that the protein expression of six signature genes were also up-regulated in glioma compared with normal brain tissues (Figure 11A–L), which is consistent with the results of differential gene expression analysis (Supplementary Sheet S3).

## 4 Discussion

The term “lower-grade gliomas” was invented to denote WHO Grade II and III oligodendrogliomas and astrocytomas, in contrast to glioblastoma. LGGs form a group of relatively independent bio-heterogeneous tumors (Picca et al., 2018). In practice, histology alone is often difficult to make a relatively accurate prognosis estimation, and tumors belonging to the same WHO grade may exhibit different malignant behaviors, depending on their molecular characteristics (Mair et al., 2021).

In recent years, it has been found that molecular subsets of LGGs could stratify patients into distinctly prognostic groups, which are superior to histological classification (Lin et al., 2020; Tu et al., 2020; Zhang et al., 2020). Meanwhile, models with reliable performance are considered valuable prognosis markers for cancers. Despite much researches have shown that CRs play a specific role in gliomas (Gusyatiner & Hegi 2018; Chung et al., 2020), its in-depth value in LGGs management was previously rarely reported.

In our current work, We first identified 149 DE-CRs. Then we comprehensively analyzed the functional enrichment for 149 CRs and constructed PPI networks. Next, we filtered 51 CRs associated with the prognosis of LGGs by univariate regression analysis. Followed by LASSO and multivariate Cox regression analysis, we constructed an efficient risk model which consisted of 14 CRs and stratified patients into high- and low-risk groups by the risk score. The good predictive performance of the CRs-related risk model was also confirmed via KM and ROC curves both in TCGA and CGGA cohorts. Subsequently, the independent predictive role of the signature was verified. Additionally, combined with other clinicopathological factors, a personalized predicted nomogram taking risk score was established to predict prognosis. The CRs-related signature is also closely related to immune cell infiltration, and tumor mutation and 10 small molecule drugs have been found, opening a new window for the management of LGGs. Finally, We verified several representative signature genes using IHC, SC-RNA sequencing analysis and RT-qPCR. We found that the expression of six representative genes was consistent at the transcriptional and protein levels. These results are in agreement with the results of the differentially expressed genes.

In this research, we identified that the CRs-related signature includes fourteen genes, nine of which have been reported in the literature (Li et al., 2013; Minchenko et al., 2014; Zhang et al., 2015; Bao et al., 2017; Chen et al., 2021; Huang et al., 2021; Li J. et al, 2022; Li SH. et al, 2022; Hu et al., 2022). Chromobox 7 (CBX7), is a member of the chromobox family. It has been reported to involve in oncogenesis, which may be deregulated in gliomas (Bao et al., 2017), breast cancer (Kim et al., 2015), cervical carcinoma (Maimaitirexiati et al., 2021), lung cancer (Yang et al., 2021), etc. Studies have shown that CBX7 can participate in maintaining the growth of a variety of normal cells and immortalizing mouse fibroblasts (Jung et al., 2019), but the controversy about its role persists. Some works of literature have reported that CBX7 is carcinogenic in several types of cancer. But other studies have found that CBX7 may play an anticancer role in some cancers. Besides, as a tumor suppressor, CBX7 is pivotal to regulate tumor invasion and migration via the Wnt/β-catenin pathway in glioma [14]. Our study found that compared with normal brain tissue, CBX7 was continuously down-regulated at the transcriptional and protein levels in LGGs tissue. Previous studies have shown that hnRNPA1, as a protein-coding gene, is up-regulated in glioma. Furthermore, the siRNA-based strategy against USP8 is effective to deal with glioma tumor reoccurrence by targeting the hnRNPA1 oncogene [13]. Yan et al. first identified IDH1 mutations in exon sequencing of gliomas (Yan et al., 2009). In 2016, WHO added molecular informatics including IDH1 mutations to the classification of central nervous system tumors on a histological basis. As one of the most common mutations in glioma cells, the R132H variant of IDH1 occurs in 80%–90% of grade II and III gliomas (Louis et al., 2016). Especially, gene-expression analysis of single cell, RT-qPCR and protein level are warranted to validate our findings, all of which indicated that it was highly expressed in tumor tissues. NAP1L1, as a marker of malignancy. It has been reported that the expression was significantly increased in gliomas, which was significantly correlated with WHO grade, KPS, Ki-67 index, and tumor recurrence (Chen et al., 2021). L-H Zhang et al. showed that TRIM24 promotes glioma cell infiltrating and enhances resistance to temozolomide (TMZ) through activation of the PI3K/Akt signaling [15]. In our research, half of these fourteen CRs (TRIM24, HMG20B, IDH1, RCC1, RYBP, ZNF541, and LBR) were related to poor prognosis, whereas the other half genes (PCGF2, CBX6, SGF29, NAP1L1, CBX7, USP49, and HNRNPA1) had the opposite effect. Our study suggests that TRIM24, IDH1, LBR, HMG20B, USP49 and RCC1 were all highly expressed in glioma tissues and gliomar cell lines both at the mRNA and protein level. These studies pointed out that the inhibition or activation of CRs has potential clinical value, and a better understanding of CRs might provide therapeutic candidates and prognostic value for LGGs.

Based on DE-CRs between the two risk groups, we executed GO and KEGG analyses. The results suggested that DE-CRs were remarkably enriched in the cell division cycle, such as histone modification, chromatin remodeling, Cell cycle, p53 signaling pathway, and so on. Histone modification can change gene expression catalyzed by histone modification enzymes, which play an important role in the pathological process of malignant tumors (Strahl & Allis 2000). An in-depth study of histone modification not only helps to understand the gene expression and regulation but also provides a new theoretical basis for the treatment of tumors. In addition, Combining the results of disease enrichment with the results of PPI core genes, we found that the TP53 gene had the highest correlation in the PPI network. TP53, a transcription factor, is the most commonly mutated gene in all human cancers (Mendiratta et al., 2021). Emerging evidence showed that the activation of TP53 leads to cell cycle arrest (Chen 2016).

CRs have been reported to be widely involved in tumorigenesis by reprogramming the microenvironment (D'Angelo et al., 2019; Griffin et al., 2021). Consistently, Our research also showed that the CRs-related signature may play a strong regulatory role in immune-based pathways. The different risk groups exhibited distinct immune landscapes. For example, in recent years, gliomas with a higher abundance of CD8 T cells have been found to respond better to immunotherapy drugs (Kane et al., 2020; Watson et al., 2021). Similarly, our study unveiled that the abundance of CD8 + T cell was lower in LGGs patients with high-risk scores. Furthermore, We also explored the TMB and the response of patients in different risk groups to several chemotherapeutic drugs. TP53, ATRX, PTEN, and EGFR evinced higher mutation rates in the high-risk group whereas NOTCH1, CIC and IDH1 had higher mutation rates in the low-risk group. Notably, IDH1 is not only the gene with the highest percentage of mutated genes, but also can be seen in 14 CRS based features. Subsequently, GSEA results showed that CRs-related signatures were mainly enriched in immune-based signaling pathways, such as the complement and coagulation cascades, cytokine cytokine receptor interaction, FC gamma R mediated phagocytosis, leukocyte transendothelial migration, and toll like receptor signaling pathway, etc. Studies focusing on immune checkpoints have shown that the expression of major targets, such as CTLA4, PDCD1LG2, PDCD1, TMIGD2, and CD274 is higher in the high-risk patients than in those with lower risk scores, which may be related to the prognosis caused by different immune landscapes.

It is important to note, however, that the present study had some inherent limitations. First, it was based on bioinformatics analysis and *in vitro* experiments, and the results still need to be validated in clinical LGG patients. Second, this study also suffered from the inherent drawback of confounding bias in time and space, which include race, region, and time period of LGG patients. Finally, The stability of the model is only verified in the CGGA database, and a large number of tests need to be made in practice.

## 5 Conclusion

In short, we demonstrate that CRs related signature may participate in the pathological process of LGGs. A risk model based on fourteen genes was constructed, which could predict the clinical prognosis of LGGs individuals. Furthermore, our research provides a new perspective for an in-depth understanding of the relation between LGGs and CRs. Considering this article is based on transcriptional level data, a further study focusing on exploring the prognostic value of 14 CR-related genes was required.

## Data Availability

Publicly available datasets were analyzed in this study. This data can be found here: The raw data of this study are derived from the TCGA database (https://portal.gdc.cancer.gov/), CGGA data portal (http://www.cgga.org.cn/) and GEO data portal (https://www.ncbi.nlm.nih.gov/geo/), which are publicly available databases.

## Abbreviations

LGGs, Low grade gliomas; CR, Chromatin regulator; TCGA, The Cancer Genome Atlas; GEO, Gene Expression Omnibus; CGGA, Chinese Glioma Genome Atlas; KEGG, Kyoto Encyclopedia of Genes and Genomes; FDR, False discovery rate; FC, Fold change; GO, Gene ontology; WHO, world health organization; LASSO, least absolute shrinkage and selection operator; K-M, Kaplan–Meier; ROC: receiver operating characteristic curve; IHC, immunohistochemistry; qRT-PCR, quantitative real-time PCR; tSNE, T-distributed Stochastic Neighbor Embedding; scRNA-seq, single cell RNA-sequencing; IDH1, isocitrate dehydrogenase 1; MGMT, O-6-methylguanine-DNA methyltransferase; DE-CRs, differentially expressed-CRs; TMB, tumor mutation burden; NHA, normal human astrocyte; DSigDB, Drug Signatures database.
