# Supplementary material for: A novel risk score model based on fourteen chromatin regulators-based genes for predicting overall survival of patients with lower-grade gliomas
Source: Front Genet. 2022 Sep 26;13:957059. doi: 10.3389/fgene.2022.957059 (PMC9554745; doi:10.3389/fgene.2022.957059)
Supplement: Supplementary file 2 [file Table1.DOCX]

**Supplemental Table 1** Gene list and coefcient

| **Id** | **Gene full names** | **Coef** |
| --- | --- | --- |
| TRIM24 | Tripartite Motif Containing 24 | 0.040758 |
| HMG20B | High Mobility Group 20B | 0.01878 |
| PCGF2 | Polycomb Group Ring Finger 2 | -0.11162 |
| CBX6 | Chromobox 6 | -0.02031 |
| SGF29 | SAGA Complex Associated Factor 29 | -0.01873 |
| IDH1 | Isocitrate Dehydrogenase (NADP(+)) 1 | 0.008829 |
| RCC1 | Regulator Of Chromosome Condensation 1 | 0.010136 |
| RYBP | RING1 And YY1 Binding Protein | 0.126936 |
| NAP1L1 | Nucleosome Assembly Protein 1 Like 1 | -0.0063 |
| ZNF541 | Zinc Finger Protein 541 | 2.698229 |
| CBX7 | Chromobox 7 | -0.0357 |
| USP49 | Ubiquitin Specific Peptidase 49 | -0.04241 |
| HNRNPA1 | Heterogeneous Nuclear Ribonucleoprotein A1 | -0.00161 |
| LBR | Lamin B Receptor | 0.030069 |
