# Supplementary figures and images for: A novel risk score model based on fourteen chromatin regulators-based genes for predicting overall survival of patients with lower-grade gliomas
Source: Front Genet. 2022 Sep 26;13:957059. doi: 10.3389/fgene.2022.957059 (PMC9554745; doi:10.3389/fgene.2022.957059)

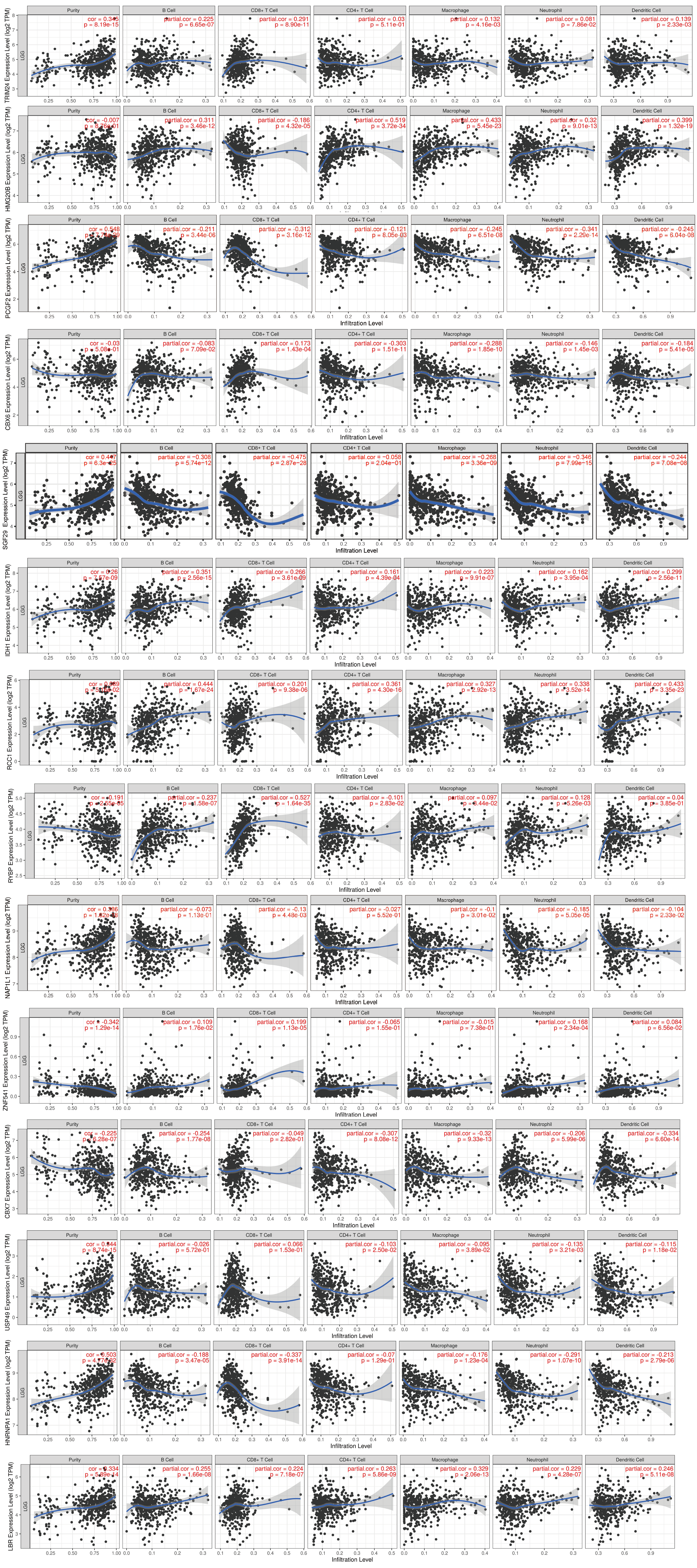

Supplement: Supplementary file 3 [file Image3.TIF]

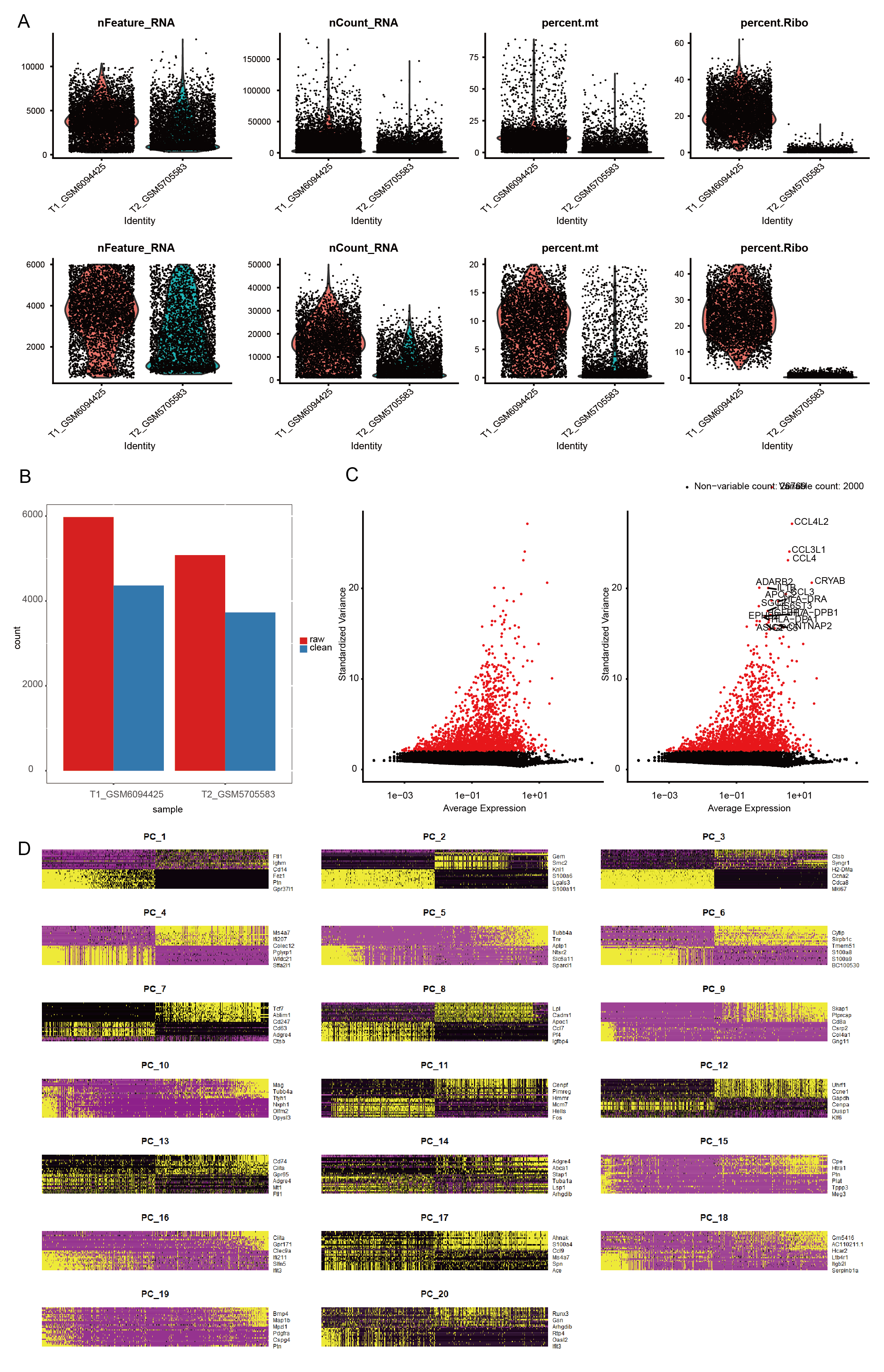

Supplement: Supplementary file 4 [file Image4.TIF]

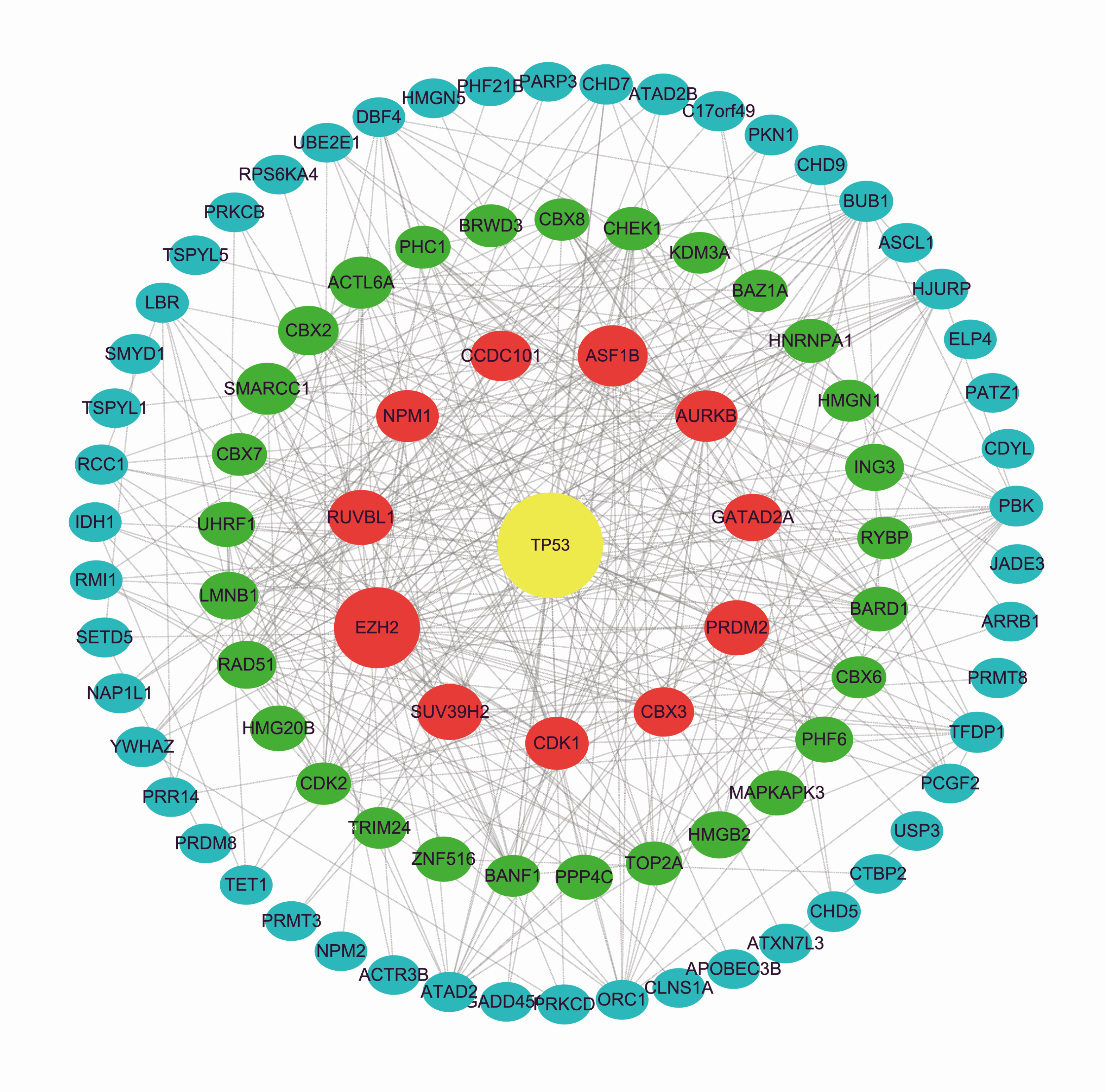

Supplement: Supplementary file 5 [file Image2.TIF]

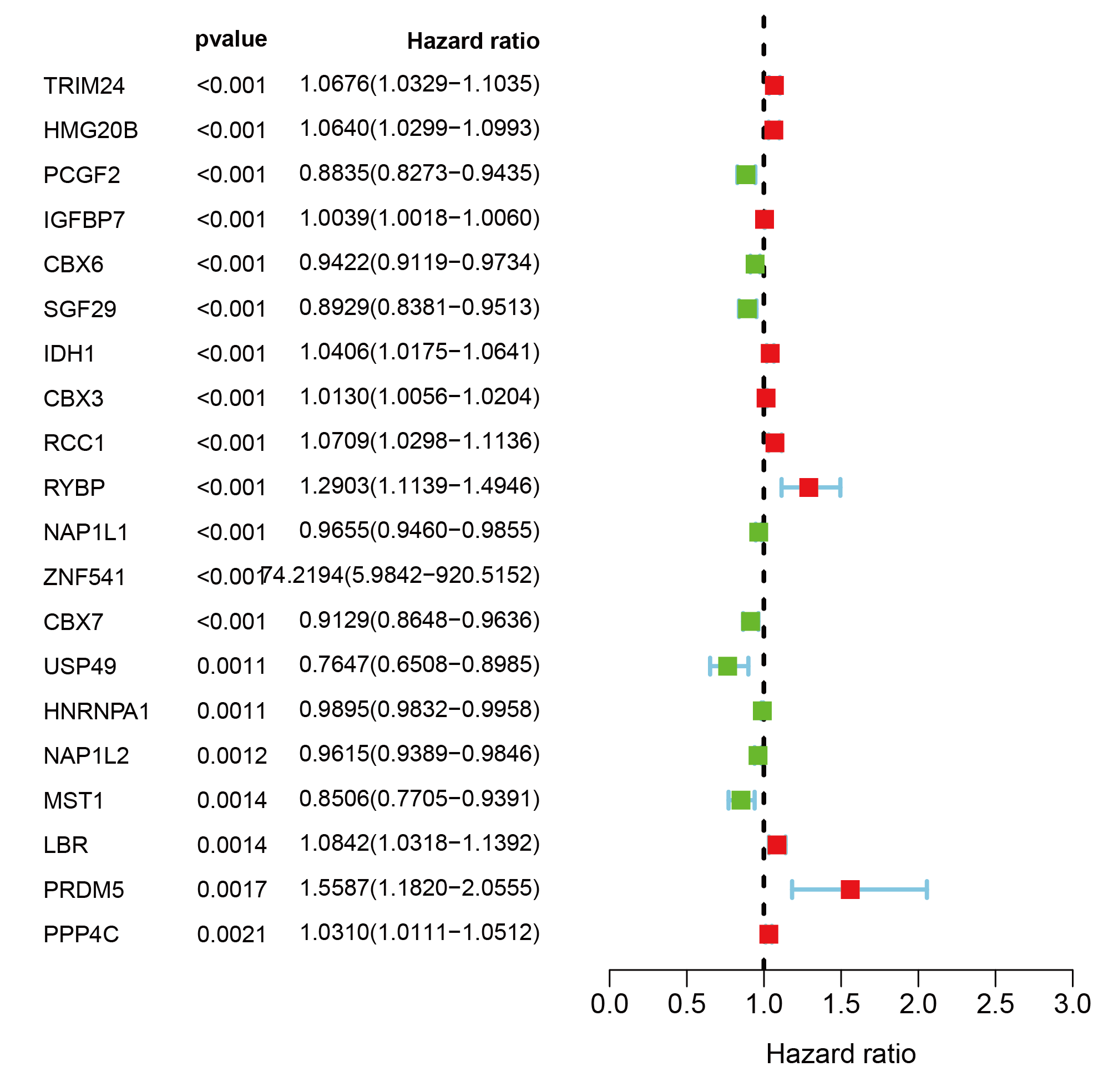

Supplement: Supplementary file 7 [file Image1.TIF]

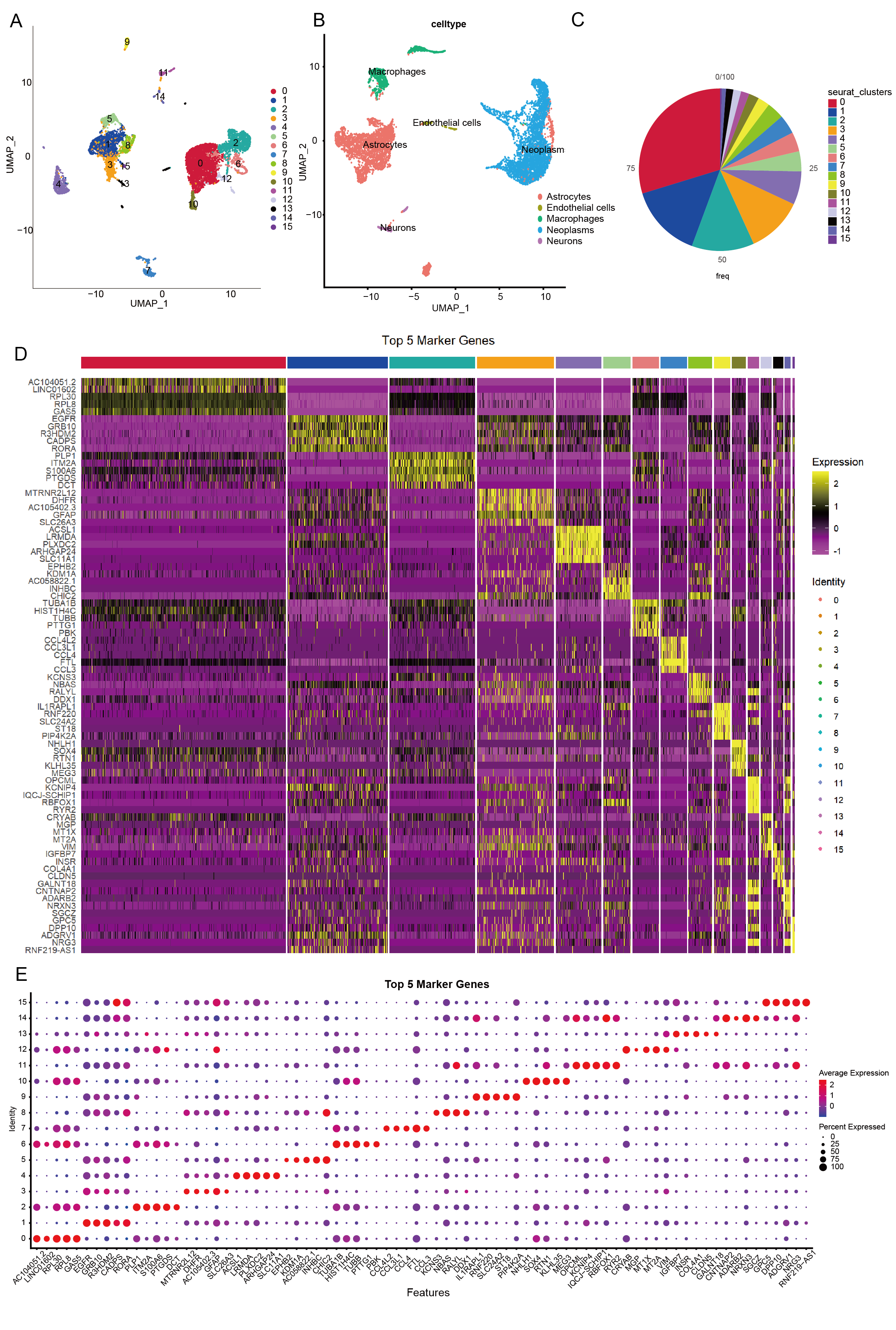

Supplement: Supplementary file 11 [file Image5.TIF]
