# Supplementary material for: A novel risk score model based on fourteen chromatin regulators-based genes for predicting overall survival of patients with lower-grade gliomas
Source: Front Genet. 2022 Sep 26;13:957059. doi: 10.3389/fgene.2022.957059 (PMC9554745; doi:10.3389/fgene.2022.957059)
Supplement: Supplementary file 6 [file Table7.DOCX]

Analysis was carried out on April 2022

Author: Zhangyongfeng

Affiliation: Department of Neurosurgery, Second Affiliated Hospital of Xi’an Jiaotong University, China.

#############################################################

Step 1: The somatic mutation and RNA-seq expression data and corresponding clinical data sheets of LGGs were obtained from the TCGA database ( https://cancergenome.nih.gov/ ) and used as the training dataset.

a：The RNA-sequencing data and related clinical information were downloaded from TCGA database (<https://www.cancer.gov/tcga>).

#metadata.cart.2022-04-30.json

#gdc_download_20220430_132808.764868.tar.gz

#gdc_download_20220425_091101.734528

b: Organizing data using perl script

use strict;

#use warnings;

use File::Copy;

my $newDir="files";

unless(-d $newDir){

mkdir $newDir or die $!;

}

opendir(RD, ".") or die $!;

my @allFiles=readdir(RD);

closedir(RD);

foreach my $subDir(@allFiles)

{

next if($subDir eq '.');

next if($subDir eq '..');

if((-d $subDir) && ($subDir ne $newDir))

{

opendir(SUB,"./$subDir") or die $!;

while(my $file=readdir(SUB))

{

if($file=~/\.tsv$/)

{

#`cp ./$subDir/$file ./$newDir`;

copy("$subDir/$file","$newDir") or die "Copy failed: $!";

}

}

close(SUB);

}

}

#############################################################

use strict;

#use warnings;

my $file=$ARGV[0];

#use Data::Dumper;

use JSON;

my $json = new JSON;

my $js;

my %hash=();

my @normalSamples=();

my @tumorSamples=();

open JFILE, "$file";

while(<JFILE>) {

$js .= "$_";

}

my $obj = $json->decode($js);

#my @samp1e=(localtime(time));

for my $i(@{$obj})

{

my $file_name=$i->{'file_name'};

my $file_id=$i->{'file_id'};

my $entity_submitter_id=$i->{'associated_entities'}->[0]->{'entity_submitter_id'};

$file_name=~s/\.gz//g;

if(-f $file_name)

{

my @idArr=split(/\-/,$entity_submitter_id);

if($idArr[3]=~/^0/)

{

push(@tumorSamples,$entity_submitter_id);

}

else

{

push(@normalSamples,$entity_submitter_id);

}

open(RF,"$file_name") or die $!;

while(my $line=<RF>)

{

next if($line=~/^\n/);

next if($line=~/^\_/);

chomp($line);

my @arr=split(/\t/,$line);

${$hash{$arr[1]}}{$entity_submitter_id}=$arr[3];

}

close(RF);

}

}

#print Dumper $obj

open(WF,">mRNAmatrix.txt") or die $!;

my $normalCount=$#normalSamples+1;

my $tumorCount=$#tumorSamples+1;

if($normalCount==0)

{

print WF "id";

}

else

{

print WF "id\t" . join("\t",@normalSamples);

}

print WF "\t" . join("\t",@tumorSamples) . "\n";

foreach my $key(keys %hash)

{

print WF $key;

foreach my $normal(@normalSamples)

{

print WF "\t" . ${$hash{$key}}{$normal};

}

foreach my $tumor(@tumorSamples)

{

print WF "\t" . ${$hash{$key}}{$tumor};

}

print WF "\n";

}

close(WF);

print "normal count: $normalCount\n";

print "tumor count: $tumorCount\n";

b: Downloading the mutation data sheets of LGGs from TCGA database.

# gdc_download_20220430_082618.017644.tar.gz

use strict;

#use warnings;

my %hash=();

open(RF, "risk.all.txt") or die $!;

while(my $line=<RF>){

chomp($line);

my @arr=split(/\t/, $line);

$hash{$arr[0]}=$arr[$#arr];

}

close(RF);

my %fieldHash=();

my $lineCount=0;

open(RF, "input.maf") or die $!;

open(LOW, ">low.maf") or die $!;

open(HIGH, ">high.maf") or die $!;

while(my $line=<RF>){

#my @samp1e=(localtime(time));

next if($line=~/^\n/);

next if($line=~/^\#/);

$lineCount++;

chomp($line);

my @arr=split(/\t/,$line);

if($lineCount==1){

for(my $i=0;$i<=$#arr;$i++){

$fieldHash{$arr[$i]}=$i;

}

print LOW $line . "\n";

print HIGH $line . "\n";

next;

}

#

if($arr[$fieldHash{"Amino_acids"}] eq ""){

next;

}

if($arr[$fieldHash{"Variant_Classification"}] eq "Silent"){

next;

}

if($arr[$fieldHash{"Variant_Classification"}] eq "Splice_Region"){

next;

}

my $sampleName=$arr[$fieldHash{"Tumor_Sample_Barcode"}];

my @sampleArr=split(/\-/, $sampleName);

my $subSampleName="$sampleArr[0]-$sampleArr[1]-$sampleArr[2]";

if(exists $hash{$subSampleName}){

if($hash{$subSampleName} eq "high"){

$arr[$fieldHash{"Tumor_Sample_Barcode"}]=$subSampleName;

print HIGH join("\t", @arr) . "\n";

}

if($hash{$subSampleName} eq "low"){

$arr[$fieldHash{"Tumor_Sample_Barcode"}]=$subSampleName;

print LOW join("\t", @arr) . "\n";

}

}

}

close(HIGH);

close(LOW);

close(RF);

#############################################################

Step 2: The CGGA mRNAseq_693 (containing 282 primary LGGs) and mRNAseq_325 (containing 144 primary LGGs) data sets were selected as the validation cohort for the Rscore.

# CGGA.mRNAseq_693.RSEM-genes.20190701.txt.zip.

# CGGA.mRNAseq_325.RSEM-genes.20190701.txt.zip

############################################################

Step 3 Statistical analysis of the pooled data from the combined genes was performed.

setwd("File path")

cr=read.table("cr.txt",header = T,sep = "\t",check.names = F)

mrna=read.table("mRNA.txt",header = T,sep = "\t",check.names=F)

chromatin_gene=merge(chromatin,mrna,by="id")

write.table(chromatin_gene,"chromatin_mRNA.txt",quote = F,row.names = F,sep = "\t")

#####################################

if (!requireNamespace("BiocManager", quietly = TRUE))

# install.packages("BiocManager")

#BiocManager::install("limma")

#install.packages("pheatmap")

#install.packages("ggplot2")

#

library(ggplot2)

library(limma)

library(pheatmap)

logFCfilter=1

adjPfilter=0.05

expFile="geneMatrix.txt"

conFile="sample1.txt"

treatFile="sample2.txt"

rt=read.table(expFile,sep="\t",header=T,check.names=F)

rt=as.matrix(rt)

rownames(rt)=rt[,1]

exp=rt[,2:ncol(rt)]

dimnames=list(rownames(exp),colnames(exp))

data=matrix(as.numeric(as.matrix(exp)),nrow=nrow(exp),dimnames=dimnames)

data=avereps(data)

#data=log2(data+1)

data=normalizeBetweenArrays(data)

sample1=read.table(conFile,sep="\t",header=F,check.names=F)

sample2=read.table(treatFile,sep="\t",header=F,check.names=F)

conData=data[,as.vector(sample1[,1])]

treatData=data[,as.vector(sample2[,1])]

rt=cbind(conData,treatData)

conNum=ncol(conData)

treatNum=ncol(treatData)

Type=c(rep("con",conNum),rep("treat",treatNum))

design <- model.matrix(~0+factor(Type))

colnames(design) <- c("con","treat")

fit <- lmFit(rt,design)

cont.matrix<-makeContrasts(treat-con,levels=design)

fit2 <- contrasts.fit(fit, cont.matrix)

fit2 <- eBayes(fit2)

allDiff=topTable(fit2,adjust='fdr',number=200000)

write.table(allDiff,file="GEO_all.xls",sep="\t",quote=F)

diffSig=allDiff[with(allDiff, (abs(logFC)>logFCfilter & adj.P.Val < adjPfilter )), ]

diffSigOut=rbind(id=colnames(diffSig),diffSig)

write.table(diffSigOut,file="GEO_diff.xls",sep="\t",quote=F,col.names=F)

write.table(diffSigOut,file="GEO_diff.txt",sep="\t",quote=F,col.names=F)

geneNum=50

diffSig=diffSig[order(as.numeric(as.vector(diffSig$logFC))),]

diffGeneName=as.vector(rownames(diffSig))

diffLength=length(diffGeneName)

hmGene=c()

if(diffLength>(geneNum*2)){

hmGene=diffGeneName[c(1:geneNum,(diffLength-geneNum+1):diffLength)]

}else{

hmGene=diffGeneName

}

hmExp=rt[hmGene,]

Type=c(rep("Control",conNum),rep("SCI",treatNum))

names(Type)=colnames(rt)

Type=as.data.frame(Type)

pdf(file="GEO_heatmap.pdf",height=8,width=10)

pheatmap(hmExp,

annotation=Type,

color = colorRampPalette(c("blue", "white", "red"))(50),

cluster_cols =F,

show_colnames = F,

scale="row",

fontsize = 8,

fontsize_row=6,

fontsize_col=8)

dev.off()

Significant=ifelse((allDiff$adj.P.Val<adjPfilter & abs(allDiff$logFC)>logFCfilter), ifelse(allDiff$logFC>logFCfilter,"Up","Down"), "Not")

p = ggplot(allDiff, aes(logFC, -log10(adj.P.Val)))+

geom_point(aes(col=Significant))+

scale_color_manual(values=c("#4DBBD5CC", "black", "#F39B7FCC"))+

labs(title = " ")+

theme(plot.title = element_text(size = 16, hjust = 0.5, face = "bold"))

p=p+theme_bw()

#####################################

Step 4: Univariate analysis.

library(survival)

dt1=read.table("diffgeneEXP.txt",header = T,sep = "\t",check.names = F)

st<- which(substr(colnames(dt1),14,15) == '11')

tumor=dt1[,-st]

nc=substr(colnames(tumor),1,12)

colnames(tumor)=nc

tumor=t(tumor)

write.table(tumor,"tumor.txt",sep = "\t",quote = F,col.names = F)

dt4=read.table("tumor.txt",header = T,sep = "\t",check.names =F)

clidata=read.table("time.txt",header = T,sep = "\t",check.names =F)

cliexp=merge(clidata,dt4,by="id")

write.table(cliexp,"timeexp.txt",sep = "\t",quote = F,row.names = F)

inputfile="timeexp.txt"

data1=read.table(inputfile,header = T,sep = "\t",check.names =F,row.names = 1)

data1$survival_time=data1$survival_time/365

coxf<-function(x){

fmla1 <- as.formula(Surv(survival_time,status)~data1[,x])

mycox <- coxph(fmla1,data=data1)

}

newdf=data.frame()

for(a in colnames(data1[,3:ncol(data1)])){

mycox=coxf(a)

coxResult = summary(mycox)

newdf=rbind(newdf,

cbind(id=a,

HR=coxResult$conf.int[,"exp(coef)"],

HR_95L=coxResult$conf.int[,"lower .95"],

HR_95U=coxResult$conf.int[,"upper .95"],

P=coxResult$coefficients[,"Pr(>|z|)"]

))

}

newdf=newdf[(newdf$P<0.05),]

write.table(newdf,"result.txt",sep="\t",row.names=F,quote=F)

###################forest################

HRdf1<- read.table("top20.txt", header=T, sep="\t", check.names=F, row.names=1)

idname <- rownames(HRdf1)

HR=sprintf("%.4f",HRdf1[,"HR"])

HR_95L=sprintf("%.4f",HRdf1[,"HR_95L"])

HR_95U=sprintf("%.4f",HRdf1[,"HR_95U"])

P=HRdf1[,"P"]

P=ifelse(P<0.001, "<0.001", sprintf("%.4f", P))

pdf("forest.pdf")

snum <- nrow(HRdf1)

snum2 <- snum+1

layout(matrix(c(1,2),nc=2),width=c(3,2.5))

par(mar=c(4,2.5,2,1))

plot(1,xlim= c(0,3),ylim=c(1,snum2),type="n",axes=F,xlab="",ylab="")

text(0,snum:1,idname,adj=0,cex=0.8)

text(1.5-0.5*0.2,snum:1,P,adj=1,cex=0.8);text(1.5-0.5*0.2,snum+1,'pvalue',cex=0.8,font=2,adj=1)

text(3.1,snum:1, paste0(HR,"(",HR_95L,"-",HR_95U,")"),adj=1,cex=0.8);text(3.1,snum+1,'Hazard ratio',cex=0.8,font=2,adj=1)

par(mar=c(4,1,2,1),mgp=c(2,0.5,0))

xlim = c(0,max(as.numeric(HR_95L),as.numeric(HR_95U)))

plot(1,xlim=c(0,3),ylim=c(1,snum2),type="n",axes=F,ylab="",xaxs="i",xlab="Hazard ratio")

arrows(as.numeric(HR_95L),snum:1,as.numeric(HR_95U),

snum:1,angle=90,code=3,length=0.05,col="skyblue",lwd=2.5)

abline(v=1,col="black",lty=2,lwd=2)

points(as.numeric(HR), snum:1, pch = 15,

col = ifelse(as.numeric(HR) > 1, "red", "green")

, cex=1.5)

axis(1)

dev.off()

#####################################

Step 5 LASSO Cox regression analysis (LASSO, least absolute shrinkage, and selection operator).

#install.packages("glmnet")

library(glmnet)

library(survival)

mydata1<-read.table("timeexp.txt",header=T,sep="\t",row.names = 1,check.names = F,stringsAsFactors = F)

mydata2=read.table("top20.txt",header=T,sep="\t",check.names=F)

mydata1=mydata1[,c("survival_time","status",as.vector(mydata2[,1]))]

mydata1$survival_time=mydata1$survival_time/365

v1<-as.matrix(mydata1[,c(3:ncol(mydata1))])

v2 <- as.matrix(Surv(mydata1$survival_time,mydata1$status))

myfit <- glmnet(v1, v2, family = "cox")

pdf("lambda.pdf")

plot(myfit, xvar = "lambda", label = TRUE)

dev.off()

myfit2 <- cv.glmnet(v1, v2, family="cox",nfolds = 10)

pdf("min.pdf")

plot(myfit2)

abline(v=log(c(myfit2$lambda.min,myfit2$lambda.1se)),lty="dashed")

dev.off()

coe <- coef(myfit, s = myfit2$lambda.min)

act_index <- which(coe != 0)

act_coe <- coe[act_index]

lassogene=row.names(coe)[act_index]

gene_coef=cbind(id=lassogene,coef=act_coe)

write.table(gene_coef,"gene_coef.txt",sep="\t",quote=F,row.names=F)

mygeneEXP=mydata1[,lassogene]

expcoef=function(x){crossprod(as.numeric(x),act_coe)}

Riskscore=apply(mygeneEXP,1,expcoef)

Riskgroup=as.vector(ifelse(Riskscore>median(Riskscore),"High","Low"))

newdf2=cbind(mydata1[,c("survival_time","status",lassogene)],Riskscore=as.vector(Riskscore),Riskgroup)

newdf3=cbind(id=rownames(newdf2),newdf2)

write.table(newdf3,"Riskscore.txt",sep="\t",quote=F,row.names=F)

#####################################

Step 6 survival analysis.

#install.packages("survminer")

library(survival)

library(survminer)

mydata=read.table("Riskscore.txt", header=T, sep="\t", check.names=F)

mydiff=survdiff(Surv(survival_time, status) ~Riskgroup,data = mydata)

myfit <- survfit(Surv(survival_time, status) ~ Riskgroup, data = mydata)

p=1-pchisq(mydiff$chisq,df=1)

if(p<0.001){

p="p<0.001"

}else{

p=paste0("p=",sprintf("%.03f",p))

}

pdf("survival_risk.pdf",onefile = FALSE,10,8)

ggsurvplot(myfit,

data=mydata,

conf.int=T,

pval=p,

pval.size=6,

legend.title="Risk",

legend.labs=c("High risk", "Low risk"),

xlab="Time(years)",

break.time.by = 1,

palette=c("red", "blue"),

risk.table=TRUE,

risk.table.title="",

risk.table.col = "strata",

risk.table.height=.25)

dev.off()

#############################################################################

#######################################riskline##############################

myrisk=read.table("Riskscore.txt",sep="\t",

header=T,row.names=1,check.names=F)

head(myrisk)

myrisk=myrisk[order(myrisk$Riskscore),]

mygroup=myrisk[,"Riskgroup"]

ll=length(mygroup[mygroup=="Low"])

hl=length(mygroup[mygroup=="High"])

lm=max(myrisk$Riskscore[mygroup=="Low"])

line=myrisk[,"Riskscore"]

line[line>10]=10

pdf("riskline.pdf",8,6)

plot(line, type="p", pch=16,

xlab="Patients (increasing risk socre)", ylab="Risk score",

col=c(rep("green",ll),rep("red",hl)) )

abline(h=lm,v=ll,lty=2)

dev.off()

pdf("riskpoint.pdf",8,6)

plot(myrisk$survival_time, pch=16,

xlab="Patients (increasing risk socre)", ylab="Survival time (years)",

col=ifelse(myrisk$status=="1","red","blue"))

legend("topright", c("Dead", "Alive"),pch=16,col=c("red","blue"),cex=1.2)

abline(v=ll,lty=2)

dev.off()

#####################################

Step 7 ROC curves were plotted by the ‘survivalROC’ package.

library(timeROC)

library(survival)

TCGA<-read.table("Riskscore.txt",header=T,sep="\t")

predict_1_year<- 1

predict_3_year<- 3

predict_5_year<- 5

predict_10_year<- 10

ROC<-timeROC(T=TCGA$survival_time,delta=TCGA$status,

marker=TCGA$Riskscore,cause=1,

weighting="marginal",

times=c(predict_1_year,predict_3_year,predict_5_year,predict_10_year),ROC=T)

pdf("ROC.pdf")

plot(ROC,time=predict_1_year,title=F,lwd=3)

plot(ROC,time=predict_3_year,col="yellow",add=T,title=F,lwd=3)

plot(ROC,time=predict_5_year,col="blue",add=T,title=F,lwd=3)

plot(ROC,time=predict_10_year,col="green",add=T,title=F,lwd=3)

legend("bottomright",

c(paste("AUC of 1 year survival: ",round(ROC$AUC[1],3)),

paste("AUC of 3 year survival: ",round(ROC$AUC[2],3)),

paste("AUC of 5 year survival: ",round(ROC$AUC[3],3)),

paste("AUC of 10 year surviva",round(ROC$AUC[4],3))),col=c("red","yellow","blue","green"),lwd=3)

dev.off()

#####################################

Step 8 The heat map was drawn based on the patient risk score.

library(pheatmap)

riskdata=read.table("Riskscore.txt",header = T,sep = "\t",row.names = 1)

riskdata=riskdata[order(riskdata$Riskscore),]

nriskdata=riskdata[c(3:(ncol(riskdata)-2))]

nriskdata=t(nriskdata)

nriskdata=log2(nriskdata+1)

mycolor=list()

mycolor2=c("green", "red")

names(mycolor2)=c("Low", "High")

mycolor[["Riskgroup"]]=mycolor2

ann=data.frame(Riskgroup=riskdata[,ncol(riskdata)])

rownames(ann)=rownames(riskdata)

pdf("heatmap.pdf",10,8)

pheatmap(nriskdata,

annotation=ann,

annotation_colors = mycolor,

cluster_cols = F,

cluster_rows = F,

show_colnames = F,

scale="row",

color= colorRampPalette(c("green", "black", "red"))(50),

fontsize_col=4,

fontsize=8,

fontsize_row=8)

dev.off()

#####################################

Step 9 The factors that were verified by univariate Cox regression analysis were entered into the multivariate Cox regression analysis.

library(survival)

data1=read.table("Riskscore.txt",header = T,sep = "\t",check.names = F)

data2<-read.table("clinical.txt",header=T,sep="\t",check.names = F)

data3=merge(data1,data2,by="id")

write.table(data3,"cr.txt",quote = F,sep = "\t",row.names = F)

#############################

ndata1=read.table("cr.txt",header = T,sep = "\t",check.names =F,row.names = 1)

coxf<-function(x){

fmla1 <- as.formula(Surv(survival_time,status)~ndata1[,x])

mycox <- coxph(fmla1,data=ndata1)

}

newdf=data.frame()

for(a in colnames(ndata1[,3:ncol(ndata1)])){

mycox=coxf(a)

coxResult = summary(mycox)

newdf=rbind(newdf,

cbind(id=a,

HR=coxResult$conf.int[,"exp(coef)"],

HR_95L=coxResult$conf.int[,"lower .95"],

HR_95U=coxResult$conf.int[,"upper .95"],

P=coxResult$coefficients[,"Pr(>|z|)"]

))

}

write.table(newdf,"result.txt",sep="\t",row.names=F,quote=F)

################################forest#############################################

HRdf1=read.table("result.txt",header=T,sep="\t",row.names=1,check.names=F)

idname <- rownames(HRdf1)

HR=sprintf("%.4f",HRdf1[,"HR"])

HR_95L=sprintf("%.4f",HRdf1[,"HR_95L"])

HR_95U=sprintf("%.4f",HRdf1[,"HR_95U"])

P=HRdf1[,"P"]

P=ifelse(P<0.001, "<0.001", sprintf("%.4f", P))

pdf("forest1.pdf",8,6)

snum <- nrow(HRdf1)

snum2 <- snum+1

layout(matrix(c(1,2),nc=2),width=c(3,2.5))

par(mar=c(4,2.5,2,1))

plot(1,xlim= c(0,3),ylim=c(1,snum2),type="n",axes=F,xlab="",ylab="")

text(0,snum:1,idname,adj=0,cex=0.8)

text(1.5-0.5*0.2,snum:1,P,adj=1,cex=0.8);text(1.5-0.5*0.2,snum+1,'pvalue',cex=0.8,font=2,adj=1)

text(3.1,snum:1, paste0(HR,"(",HR_95L,"-",HR_95U,")"),adj=1,cex=0.8);text(3.1,snum+1,'Hazard ratio',cex=0.8,font=2,adj=1)

par(mar=c(4,1,2,1),mgp=c(2,0.5,0))

xlim = c(0,max(as.numeric(HR_95L),as.numeric(HR_95U)))

plot(1,xlim=c(0,3),ylim=c(1,snum2),type="n",axes=F,ylab="",xaxs="i",xlab="Hazard ratio")

arrows(as.numeric(HR_95L),snum:1,as.numeric(HR_95U),

snum:1,angle=90,code=3,length=0.05,col="skyblue",lwd=2.5)

abline(v=1,col="black",lty=2,lwd=2)

points(as.numeric(HR), snum:1, pch = 15,

col ="green"

, cex=1.5)

axis(1)

dev.off()

##########################################Cox###################################

fmla2 <- as.formula(Surv(survival_time,status)~.)

mycox2 <- coxph(fmla2,data=ndata1)

coxResult2=summary(mycox2)

newdf2=cbind(

HR=coxResult2$conf.int[,"exp(coef)"],

HR_95L=coxResult2$conf.int[,"lower .95"],

HR_95U=coxResult2$conf.int[,"upper .95"],

P=coxResult2$coefficients[,"Pr(>|z|)"])

newdf2=cbind(id=row.names(newdf2),newdf2)

write.table(newdf2,"result2.txt",sep="\t",row.names=F,quote=F)

##################forest#############################################################

HRdf1=read.table("result2.txt",header=T,sep="\t",row.names=1,check.names=F)

idname <- rownames(HRdf1)

HR=sprintf("%.4f",HRdf1[,"HR"])

HR_95L=sprintf("%.4f",HRdf1[,"HR_95L"])

HR_95U=sprintf("%.4f",HRdf1[,"HR_95U"])

P=HRdf1[,"P"]

P=ifelse(P<0.001, "<0.001", sprintf("%.4f", P))

pdf("forest2.pdf",8,6)

snum <- nrow(HRdf1)

snum2 <- snum+1

layout(matrix(c(1,2),nc=2),width=c(3,2.5))

par(mar=c(4,2.5,2,1))

plot(1,xlim= c(0,3),ylim=c(1,snum2),type="n",axes=F,xlab="",ylab="")

text(0,snum:1,idname,adj=0,cex=0.8)

text(1.5-0.5*0.2,snum:1,P,adj=1,cex=0.8);text(1.5-0.5*0.2,snum+1,'pvalue',cex=0.8,font=2,adj=1)

text(3.1,snum:1, paste0(HR,"(",HR_95L,"-",HR_95U,")"),adj=1,cex=0.8);text(3.1,snum+1,'Hazard ratio',cex=0.8,font=2,adj=1)

par(mar=c(4,1,2,1),mgp=c(2,0.5,0))

xlim = c(0,max(as.numeric(HR_95L),as.numeric(HR_95U)))

plot(1,xlim=c(0,3),ylim=c(1,snum2),type="n",axes=F,ylab="",xaxs="i",xlab="Hazard ratio")

arrows(as.numeric(HR_95L),snum:1,as.numeric(HR_95U),

snum:1,angle=90,code=3,length=0.05,col="skyblue",lwd=2.5)

abline(v=1,col="black",lty=2,lwd=2)

points(as.numeric(HR), snum:1, pch = 15,

col = "red"

, cex=1.5)

axis(1)

dev.off()

#####################################

Step10 The Wilcoxon rank sum test was used to determine correlation of clinical features between the high and low risk groups.

risk=read.table("Riskscore.txt",header = T,sep = "\t")

clinical=read.table("clinical.txt",header = T,sep = "\t")

riskcli=merge(risk,clinical,by="id")

write.table(riskcli,"riskcli.txt",quote = F,sep = "\t",row.names = F)

mykf=read.table("riskcli.txt",header=T,sep="\t",check.names=F)

head(mykf)

group1="Riskscore"

group2="pathologic_N" #

kfresult=mykf[,c(group1,group2)]

mytable=table(kfresult)

chisq.test(mytable)

mytable

fisher.test(mytable)

#####################################

Step11 In order to visualize the correlations between the risk score values of the different samples, heatmaps of the matrix of Pearson correlations between the samples were plotted using the ‘heatmap.2’.

library(pheatmap)

data1=read.table("Riskscore.txt",header = T,sep = "\t",check.names = F)

data2=read.table("clinical.txt",header = T,sep = "\t",check.names = F)

data3=merge(data1,data2,by="id")

write.table(data3,"clinicalheat.txt",sep = "\t",quote = F,row.names = F)

ht=read.table("geneheat.txt",sep="\t",header=T,

row.names=1,check.names=F)

ht=t(ht)

Group=read.table("clinicalheat.txt",sep="\t",

header=T,row.names=1,check.names=F)

Group=Group[order(Group$Riskscore),]

ht=ht[,row.names(Group)]

pdf("heatmap.pdf",10,8)

pheatmap(ht, annotation=Group,

color = colorRampPalette(c("green", "white", "red"))(50),

cluster_cols =F,

fontsize=7,

fontsize_row=8,

scale="row",

show_colnames=F,

fontsize_col=3)

dev.off()

#####################################

Step12 Differences clinical characteristics between high and low groups were analysed with the Wilcoxon rank sum test.

data1=read.table("Riskscore.txt",header = T,sep = "\t",check.names = F)

data2=read.table("clinical.txt",header = T,sep = "\t",check.names = F)

data3=merge(data1,data2,by="id")

for (a in colnames(data3[,3:ncol(data3)])){

scorename="Riskscore"

clinical=a

riskscore=data3

head(riskscore)

riskscore=riskscore[,c("id",clinical,scorename)]

colnames(riskscore)=c("id","clinical","score")

xlabel=vector()

tab1=table(riskscore[,"clinical"])

labn=length(tab1)

for(i in 1:labn ){

xlabel=c(xlabel,names(tab1[i]))

}

mytest<-wilcox.test(score ~ clinical, data = riskscore)

p=mytest$p.value

if(p<0.001){

p="p<0.001"

}else{

p=paste0("p=",sprintf("%.03f",p))

}

mybox = boxplot(score ~ clinical, data = riskscore,outline = F, plot=F)

ymin=min(mybox$stats)

ymax = max(mybox$stats/5+mybox$stats)

y1 = max(mybox$stats/10+mybox$stats)

y12 = max(mybox$stats/12+mybox$stats)

n = ncol(mybox$stats)

pdffile=paste(clinical,".pdf",sep="")

pdf(file=pdffile,8,8)

par(mar = c(4,7,3,3))

boxplot(score ~ clinical, data = riskscore,names=xlabel,xlab = "",main=clinical,

ylab = paste(scorename),col=c("#4DBBD5CC","#F39B7FCC"),

cex.main=1.6, cex.lab=1.4, cex.axis=1.3,ylim=c(ymin,ymax),outline = F)

segments(1,y1, n,y1);

segments(1,y1, 1,y12)

segments(n,y1, n,y12)

text((1+n)/2,y1,labels=p,cex=1.5,pos=3)

dev.off()

}

#####################################

Step13 Sub-group analysis was performed between high and low risk groups.

#install.packages("survminer")

library(survival)

library(survminer)

mydata1=read.table("Riskscore.txt", header=T, sep="\t", check.names=F)

mydata2=read.table("clinical.txt", header=T, sep="\t", check.names=F)

mydata3=merge(mydata1,mydata2,by="id")

head(mydata3)

clinical1="sex"

clinical2="F"

mytitle=paste0(clinical2," ","Risk")

surdata=mydata3[mydata3[,clinical1]==clinical2,]

mydiff=survdiff(Surv(survival_time, status) ~Riskgroup,data = surdata)

myfit <- survfit(Surv(survival_time, status) ~Riskgroup, data = surdata)

p=1-pchisq(mydiff$chisq,df=1)

if(p<0.001){

p="p<0.001"

}else{

p=paste0("p=",sprintf("%.03f",p))

}

pdf(file=paste0("survival.",clinical1,"2","_",".pdf"), onefile = F,8,6)

ggsurvplot(myfit,

data=surdata,

conf.int=F,

pval=p,

pval.size=6,

legend.title=mytitle,

legend.labs=c("High risk", "Low risk"),

xlab="Time(years)",

break.time.by = 1,

palette=c("red", "blue"),

risk.table=F,

)

dev.off()

#####################################

Step14 A nomogram for predicting riskscore was established, and the accuracy of this nomogram was quantified using Harrell’s concordance index (C-index).

install.packages("regplot")

#install.packages("rms")

library(survival)

library(regplot)

library(rms)

data1=read.table("cr.txt",header = T,sep = "\t",check.names =F,row.names = 1)

#nomogram

nomcox=coxph(Surv(survival_time, status) ~ . , data = data1)

regplot(nomcox,

plots = c("bars", "boxes"),

clickable=F,

title=NULL,

points=T,

droplines=T,

observation=data1[20,],

rank="sd",

failtime = c(1,3,5,10),

prfail = F)

head(data1)

pdf("calibration.pdf",10,8)

#1 year

mx1 <- cph(Surv(survival_time, status) ~Age +Gender+ Grade+ IDH1+ Riskgroup, x=T, y=T, surv=T, data=data1, time.inc=1)

cal1 <- calibrate(mx1, cmethod="KM", method="boot", u=1, m=(nrow(data1)/3), B=1000)

plot(cal1, xlim=c(0,1), ylim=c(0,1),

xlab="Nomogram-predicted Overall survival (%)", ylab="Observed Overall survival (%)", lwd=1.5, col="green", sub=F)

#3 year

mx2 <- cph(Surv(survival_time, status) ~Age +Gender+ Grade+ IDH1+ Riskgroup, x=T, y=T, surv=T, data=data1, time.inc=3)

cal2 <- calibrate(mx2, cmethod="KM", method="boot", u=3, m=(nrow(data1)/3), B=1000)

plot(cal2, xlim=c(0,1), ylim=c(0,1), xlab="", ylab="", lwd=1.5, col="blue", sub=F, add=T)

#5 year

mx3 <- cph(Surv(survival_time, status) ~Age +Gender+ Grade+ IDH1+ Riskgroup, x=T, y=T, surv=T, data=data1, time.inc=5)

cal3 <- calibrate(mx3, cmethod="KM", method="boot", u=5, m=(nrow(data1)/3), B=1000)

plot(cal3, xlim=c(0,1), ylim=c(0,1), xlab="", ylab="", lwd=1.5, col="red", sub=F, add=T)

#5 year

mx4 <- cph(Surv(survival_time, status) ~Age +Gender+ Grade+ IDH1+ Riskgroup, x=T, y=T, surv=T, data=data1, time.inc=10)

cal4 <- calibrate(mx4, cmethod="KM", method="boot", u=10, m=(nrow(data1)/3), B=1000)

plot(cal4, xlim=c(0,1), ylim=c(0,1), xlab="", ylab="", lwd=1.5, col="yellow", sub=F, add=T)

legend('bottomright', c('1-year', '3-year', '5-year','10-year'),

col=c("green","blue","red","yellow"), lwd=1.5, bty = 'n')

dev.off()

#####################################

Step15 GO and KEGG enrichment analysis GO (http://www.geneontology.org/)

#install packages

if (!requireNamespace("BiocManager", quietly = TRUE))

install.packages("BiocManager")

BiocManager::install("org.Hs.eg.db")

if (!requireNamespace("BiocManager", quietly = TRUE))

install.packages("BiocManager")

BiocManager::install("clusterProfiler")

if (!requireNamespace("BiocManager", quietly = TRUE))

install.packages("BiocManager")

BiocManager::install("enrichplot")

#library r packages

library(org.Hs.eg.db)

library(clusterProfiler)

library(enrichplot)

library(ggplot2)

gene_symbol=read.table("diffgene.txt",sep="\t",check.names=F,header=T)

gene_name=as.vector(gene_symbol[,1])

id=as.data.frame(gene_name)

geneID <- mget(gene_name, org.Hs.egSYMBOL2EG, ifnotfound=NA)

geneID <- as.character(geneID)

data=cbind(gene_symbol,entrezID=geneID)

write.table(id,"id.txt",sep="\t",quote = F,row.names = F,col.names = F)

write.table(data,"name_id.txt",sep="\t",quote = F,row.names = F)

#GO

go <- enrichGO(gene=data$entrezID,

OrgDb = org.Hs.eg.db, ont='ALL',pvalueCutoff = 0.05)#MF CC BP

write.csv(go,"go.csv",row.names =F)

pdf("GO1.pdf",12,10)

barplot(go, drop = T, showCategory =5,split="ONTOLOGY") + facet_grid(ONTOLOGY~., scale='free')

dev.off()

pdf("GO2.pdf",10,8)

dotplot(go,showCategory = 5,split="ONTOLOGY")+ facet_grid(ONTOLOGY~., scale='free')

dev.off()

####

##KEGG

kegg <- enrichKEGG(gene = data$entrezID,organism ="human",pvalueCutoff = 0.05)

write.csv(kegg,"KEGG.csv",row.names =F)

pdf("kegg1.pdf",10,8)

barplot(kegg, showCategory =10)

dev.off()

pdf("kegg2.pdf",10,8)

dotplot(kegg,showCategory = 10)

dev.off()

#####################################

Step16 Gene-set enrichment analysis (GSEA) was performed using GSEA software ( http://www.broad.mit.edu/gsea ).

#install.packages("ggplot2")

library(plyr)

library(ggplot2)

library(grid)

library(gridExtra)

setwd("C:\\Users\\lexb4\\Desktop\\CGGA\\18.multipleGSEA")

files=grep(".xls",dir(),value=T)

data = lapply(files,read.delim)

names(data) = files

dataSet = ldply(data, data.frame)

dataSet$pathway = gsub(".xls","",dataSet$.id)

gseaCol=c("#58CDD9","#7A142C","#5D90BA","#431A3D","#91612D","#6E568C","#E0367A","#D8D155","#64495D","#7CC767","#223D6C","#D20A13","#FFD121","#088247","#11AA4D")

pGsea=ggplot(dataSet,aes(x=RANK.IN.GENE.LIST,y=RUNNING.ES,colour=pathway,group=pathway))+

geom_line(size = 1.5) + scale_color_manual(values = gseaCol[1:nrow(dataSet)]) +

labs(x = "", y = "Enrichment Score", title = "") + scale_x_continuous(expand = c(0, 0)) +

scale_y_continuous(expand = c(0, 0),limits = c(min(dataSet$RUNNING.ES - 0.02), max(dataSet$RUNNING.ES + 0.02))) +

theme_bw() + theme(panel.grid = element_blank()) + theme(panel.border = element_blank()) + theme(axis.line = element_line(colour = "black")) + theme(axis.line.x = element_blank(),axis.ticks.x = element_blank(),axis.text.x = element_blank()) +

geom_hline(yintercept = 0) + theme(legend.position = c(0,0),legend.justification = c(0,0)) + #

guides(colour = guide_legend(title = NULL)) + theme(legend.background = element_blank()) + theme(legend.key = element_blank())+theme(legend.key.size=unit(0.5,'cm'))

pGene=ggplot(dataSet,aes(RANK.IN.GENE.LIST,pathway,colour=pathway))+geom_tile()+

scale_color_manual(values = gseaCol[1:nrow(dataSet)]) +

labs(x = "high expression<----------->low expression", y = "", title = "") +

scale_x_discrete(expand = c(0, 0)) + scale_y_discrete(expand = c(0, 0)) +

theme_bw() + theme(panel.grid = element_blank()) + theme(panel.border = element_blank()) + theme(axis.line = element_line(colour = "black"))+

theme(axis.line.y = element_blank(),axis.ticks.y = element_blank(),axis.text.y = element_blank())+ guides(color=FALSE)

gGsea = ggplot_gtable(ggplot_build(pGsea))

gGene = ggplot_gtable(ggplot_build(pGene))

maxWidth = grid::unit.pmax(gGsea$widths, gGene$widths)

gGsea$widths = as.list(maxWidth)

gGene$widths = as.list(maxWidth)

dev.off()

pdf('multipleGSEA.pdf',

width=7,

height=5.5)

par(mar=c(5,5,2,5))

grid.arrange(arrangeGrob(gGsea,gGene,nrow=2,heights=c(.8,.3)))

dev.off()

#####################################

Step17 Correlation analysis was then conducted to explore the 14-gene signature’s ability to predict immune checkpoint expression and immune cell infiltration.

library(limma)

library(pheatmap)

tcga<-read.table("All_infiltration_estimation.csv",header = T,sep = ",",check.names = F)

tcga=as.matrix(tcga)

rownames(tcga)=tcga[,1]

GeneExp=tcga[,2:ncol(tcga)]

TCGA=matrix(as.numeric(as.matrix(GeneExp)),nrow=nrow(GeneExp),dimnames=list(rownames(GeneExp),colnames(GeneExp)))

nc=substr(rownames(TCGA),1,12)

rownames(TCGA)=nc

TCGA=avereps(TCGA)

tumor=cbind(id=row.names(TCGA),TCGA)

risk=read.table("Riskscore.txt",header = T,sep = "\t",check.names = F)

risk_mRNA=merge(risk,tumor,by="id")

rownames(risk_mRNA)=risk_mRNA$id

risk_mRNA=risk_mRNA[,-1]

myresult=data.frame()

mygroup=c("Riskscore")

for(i in colnames(risk_mRNA)[2:ncol(risk_mRNA)]){

mytest=wilcox.test(as.numeric(risk_mRNA[,i]) ~ risk_mRNA[,"Riskscore"])

p=mytest$p.value

if(p<0.05){

myresult=rbind(myresult,cbind(id=i, p))

mygroup=c(mygroup, i)

}

}

write.table(myresult,"myresult.txt" ,sep="\t", quote=F, row.names=F)

ht=risk_mRNA[,mygroup]

ht=ht[order(ht[,"Riskscore"]),]

cann=ht[,1,drop=F]

cann[,"Riskscore"]=factor(cann[,"Riskscore"], unique(cann[,"Riskscore"]))

ht=t(ht[,(2:ncol(ht))])

rann=sapply(strsplit(rownames(ht),"_"), '[', 2)

rann=as.data.frame(rann)

row.names(rann)=row.names(ht)

colnames(rann)=c("Methods")

rann[,"Methods"]=factor(rann[,"Methods"], unique(rann[,"Methods"]))

cgap=as.vector(cumsum(table(cann[,"Riskscore"])))

rgap=as.vector(cumsum(table(rann[,"Methods"])))

mycolor=rainbow(9)

mycolor=mycolor[1:length(unique(cann[,"Riskscore"]))]

Riskscore=mycolor

names(Riskscore)=levels(factor(cann[,"Riskscore"]))

colorslist=list(Riskscore=Riskscore)

ht=matrix(as.numeric(as.matrix(ht)),nrow=nrow(ht),

dimnames=list(rownames(ht),colnames(ht)))

pdf("immheatmap.pdf", 10,8)

pheatmap(ht,

annotation=cann,

annotation_row=rann,

annotation_colors = colorslist,

color = colorRampPalette(c(rep("blue",5), "white", rep("red",5)))(100),

cluster_cols =F,

cluster_rows =F,

gaps_row=rgap,

gaps_col=cgap,

scale="row",

show_colnames=F,

show_rownames=T,

fontsize=6,

fontsize_row=5,

fontsize_col=6)

dev.off()

#####################################

Step18 The 50% inhibiting concentration (IC 50 ) values were calculated using linear regression analysis and IC 50 values were considered to indicate the drug sensitivity, where low IC 50 values indicate high drug sensitivity and high IC 50 values indicate low drug sensitivity .

if (!requireNamespace("BiocManager", quietly = TRUE))

install.packages("BiocManager")

BiocManager::install(c("car", "ridge", "preprocessCore", "genefilter", "sva"))

install.packages("ggpubr")

library(car)

####

BiocManager::install(c('sva', 'car', 'genefilter', 'preprocessCore', 'ridge'))

lidocaine-4421

pseudopelletierine-2766

dapsone-1868

trazodone-2379

rottlerin-914

PF-00875133-00-5967

troleandomycin-1885

library(limma)

library(ggpubr)

library(pRRophetic)

library(ggplot2)

set.seed(666)

install.packages("preprocessCore")

setwd("D:\\Desk\\ing\\try again\\22")

#mydrugs=c(")

mydrugs=c("Cyclopamine", "Cytarabine", "Dasatinib", "DMOG", "Docetaxel")

risk=read.table("Riskscore.txt",header = T,sep = "\t",check.names = F,row.names = 1)

mRNA=read.table("tumor.txt",header = T,sep = "\t",check.names =F)

mRNA=as.matrix(mRNA)

rownames(mRNA)=mRNA[,1]

Geneexp=mRNA[,2:ncol(mRNA)]

newdf=matrix(as.numeric(as.matrix(Geneexp)),nrow=nrow(Geneexp),dimnames=list(rownames(Geneexp),colnames(Geneexp)))

newdf=avereps(newdf)

newdf=t(newdf)

for (drugi in mydrugs){

drug_sen=pRRopheticPredict(newdf, drugi, selection=1)

drug_sen=drug_sen[drug_sen!="NaN"]

coid=intersect(row.names(risk), names(drug_sen))

risk=risk[coid, "Risk",drop=F]

drug_sen=drug_sen[coid]

drugrisk=cbind(risk, drug_sen)

risk$Risk=factor(risk$Risk, levels=c("Low", "High"))

mygroup=levels(factor(risk[,"Risk"]))

mycom=combn(mygroup, 2)

mycomlist=list()

for(b in 1:ncol(mycom)){mycomlist[[b]]<-mycom[,b]}

wilcox_test=wilcox.test(drug_sen~Risk, drugrisk)

if (wilcox_test$p.value<0.05){

myboxplot=ggboxplot(drugrisk, x="Risk", y="drug_sen", fill = "Risk", palette = c('green','red'),ylab=paste0(drugi, " senstivity (IC50)"),

add.params = list(fill="white"),

order=mygroup)+

stat_compare_means(comparisons = mycomlist)

pdf(file=paste0(drugi, ".pdf"))

print(myboxplot)

dev.off()

}

}

#####################################

Step19 Tumor Mutational Burden analysis was carried out using the Oncomine Tumor Mutation Load Assay (Thermo Fischer Scientific).

#if (!require("BiocManager"))

# install.packages("BiocManager")

#BiocManager::install("maftools")

library(maftools)

risk=read.table("risk.all.txt", header=T, sep="\t", check.names=F)

outTab=risk[,c(1, ncol(risk))]

colnames(outTab)=c("Tumor_Sample_Barcode", "Risk")

write.table(outTab, file="ann.txt", sep="\t", quote=F, row.names=F)

geneNum=15

geneMut=read.table("geneMut.txt", header=T, sep="\t", check.names=F, row.names=1)

gene=row.names(geneMut)[1:geneNum]

ann_colors=list()

col=c("blue", "red")

names(col)=c("low", "high")

ann_colors[["Risk"]]=col

pdf(file="low.pdf", width=6, height=6)

maf=read.maf(maf="low.maf", clinicalData="ann.txt")

oncoplot(maf=maf, clinicalFeatures="Risk", genes=gene, annotationColor=ann_colors, keepGeneOrder=T)

dev.off()

pdf(file="high.pdf", width=6, height=6)

maf=read.maf(maf="high.maf", clinicalData="ann.txt")

oncoplot(maf=maf, clinicalFeatures="Risk", genes=gene, annotationColor=ann_colors, keepGeneOrder=T)

dev.off()

#if (!require("BiocManager"))

# install.packages("BiocManager")

#BiocManager::install("maftools")

library(maftools)

setwd("C:\\Users\\lexb4\\Desktop\\tcgaTMB\\03.maftools")

maf = read.maf(maf = 'input.maf')

pdf(file="summary.pdf",width=7,height=6)

plotmafSummary(maf = maf, rmOutlier = TRUE, addStat = 'median', dashboard = TRUE, titvRaw = FALSE)

dev.off()

pdf(file="waterfall.pdf",width=7,height=6)

oncoplot(maf = maf, top = 30, fontSize = 12 ,showTumorSampleBarcodes = F )

dev.off()

pdf(file="interaction.pdf",width=7,height=6)

somaticInteractions(maf = maf, top = 25, pvalue = c(0.05, 0.001))

dev.off()

pdf(file="Genecloud.pdf",width=7,height=6)

geneCloud(input = maf, minMut = 5)

dev.off()

library(survival)

library(survminer)

tmbFile="TMB.txt"

riskFile="risk.all.txt"

setwd("C:\\biowolf\\cuproptosis\\33.tmbSur")

risk=read.table(riskFile, header=T, sep="\t", check.names=F, row.names=1)

tmb=read.table(tmbFile, header=T, sep="\t", check.names=F, row.names=1)

sameSample=intersect(row.names(tmb), row.names(risk))

tmb=tmb[sameSample,,drop=F]

risk=risk[sameSample,,drop=F]

data=cbind(risk, tmb)

res.cut=surv_cutpoint(data, time = "futime", event = "fustat", variables =c("TMB"))

cutoff=as.numeric(res.cut$cutpoint[1])

tmbType=ifelse(data[,"TMB"]<=cutoff, "L-TMB", "H-TMB")

scoreType=ifelse(data$risk=="low", "low risk", "high risk")

mergeType=paste0(tmbType, "+", scoreType)

bioSurvival=function(surData=null, outFile=null){

diff=survdiff(Surv(futime, fustat) ~ group, data=surData)

length=length(levels(factor(surData[,"group"])))

pValue=1-pchisq(diff$chisq, df=length-1)

if(pValue<0.001){

pValue="p<0.001"

}else{

pValue=paste0("p=",sprintf("%.03f",pValue))

}

fit <- survfit(Surv(futime, fustat) ~ group, data = surData)

#print(surv_median(fit))

bioCol=c("#FF0000","#0066FF","#6E568C","#7CC767","#223D6C","#D20A13","#FFD121","#088247","#11AA4D")

bioCol=bioCol[1:length]

surPlot=ggsurvplot(fit,

data=surData,

conf.int=F,

pval=pValue,

pval.size=6,

legend.title="",

legend.labs=levels(factor(surData[,"group"])),

font.legend=10,

legend = c(0.8, 0.8),

xlab="Time(years)",

break.time.by = 1,

palette = bioCol,

surv.median.line = "hv",

risk.table=F,

cumevents=F,

risk.table.height=.25)

pdf(file=outFile, onefile = FALSE, width=5.5, height=4.8)

print(surPlot)

dev.off()

}

data$group=tmbType

bioSurvival(surData=data, outFile="TMB.survival.pdf")

data$group=mergeType

bioSurvival(surData=data, outFile="TMB-risk.survival.pdf")

#####################################

Step 20 Code used for single cell RNA-Seq analysis is available at <https://github.com/Castelo-Branco-lab/GeneFocus>.

setwd('F:\\wtl\\work\\scRNA_001\\20210922\\02.quality')

options(stringsAsFactors = F)

library(Seurat)

library(dplyr)

library(ggplot2)

library(magrittr)

library(gtools)

library(stringr)

library(Matrix)

library(tidyverse)

library(patchwork)

library(data.table)

library(RColorBrewer)

library(ggpubr)

dir_name=c('T1','T2')

datalist=list()

for (i in 1:length(dir_name)){

dir.10x = paste0("/",dir_name[i])

my.data <- Read10X(data.dir = dir.10x)

datalist[[i]]=CreateSeuratObject(counts = my.data, project = dir_name[i],

min.cells = 3, min.features = 250)

}

names(datalist)=dir_name

for (i in 1:length(datalist)){

sce <- datalist[[i]]

sce[["percent.mt"]] <- PercentageFeatureSet(sce, pattern = "^MT-")#

sce[["percent.Ribo"]] <- PercentageFeatureSet(sce, pattern = "^RP[SL]")#

datalist[[i]] <- sce

rm(sce)

}

violin=list()

for (i in 1:length(datalist)){

violin[[i]] <- VlnPlot(datalist[[i]],

features = c("nFeature_RNA", "nCount_RNA", "percent.mt","percent.Ribo"),

pt.size = 0.1,

ncol = 4)

}

pearplot_befor <- CombinePlots(plots = violin , nrow=length(datalist), legend="none")

pearplot_befor

ggsave(filename = 'QC_before.pdf',plot = pearplot_befor,he=15,wi=15)

sce <- merge(datalist[[1]],y=datalist[2:length(datalist)])

raw_count <- table(sce@meta.data$orig.ident)

table(sce@meta.data$orig.ident)

pearplot_befor1<-VlnPlot(sce,

features = c("nFeature_RNA", "nCount_RNA", "percent.mt","percent.Ribo"),

pt.size = 0.1,

ncol = 4)

pearplot_befor1

ggsave(filename = 'QC_before1.pdf',plot = pearplot_befor1,he=7,wi=15)

rm(sce)

datalist <- lapply(X = datalist, FUN = function(x) {

x<-subset(x,subset = nFeature_RNA > 500 &

nFeature_RNA < 6000 &

quantile(percent.mt, 0.98) > percent.mt & percent.mt < 20 &

quantile(percent.Ribo, 0.99) > percent.Ribo & percent.Ribo > quantile(percent.Ribo, 0.01) &

nCount_RNA < quantile(nCount_RNA, 0.97) & nCount_RNA > 1000 )

})

sce <- merge(datalist[[1]],y=datalist[2:length(datalist)])

clean_count <- table(sce@meta.data$orig.ident)

table(sce@meta.data$orig.ident)

summary_cells <- as.data.frame(cbind(raw_count,clean_count))

counts <- rbind(as.data.frame(cbind(summary_cells[,1],rep("raw",each = length(summary_cells[,1])))),

as.data.frame(cbind(summary_cells[,2],rep("clean",each = length(summary_cells[,2])))))

counts$sample <- rep(rownames(summary_cells),times =2)

colnames(counts)<- c("count","Stat","sample")

counts[,1] <- as.numeric(counts[,1])

counts$Stat <- factor(counts$Stat, levels=c("raw", "clean"), ordered=TRUE)

fit_cell_count <- ggplot(data =counts, mapping = aes(x = sample, y=count))+

geom_bar(aes(fill = Stat),stat = 'identity', position = 'dodge') + scale_fill_brewer(palette = "Set1") +

theme(text=element_text(size=10),legend.title=element_blank(),

panel.background = element_rect(fill = "white", colour = "black",size = 0.2),

legend.key = element_rect(fill = "white", colour = "white"),

legend.background = (element_rect(colour= "white",fill = "white")))

fit_cell_count

ggsave(filename = 'fit_cell_count.pdf',plot = fit_cell_count,width = 9,height = 9)

violin_after=list()

for (i in 1:length(datalist)){

violin_after[[i]] <- VlnPlot(datalist[[i]],

features = c("nFeature_RNA", "nCount_RNA", "percent.mt","percent.Ribo"),

pt.size = 0.1,

ncol = 4)

}

pearplot_after <- CombinePlots(plots = violin_after , nrow=length(datalist), legend="none")

pearplot_after

ggsave(filename = 'QC_after.pdf',plot = pearplot_after,he=15,wi=15)

pearplot_after1 <- VlnPlot(sce,

features = c("nFeature_RNA", "nCount_RNA", "percent.mt","percent.Ribo"),

pt.size = 0.1,

ncol = 4)

pearplot_after1

ggsave(filename = 'QC_after1.pdf',plot = pearplot_after1,he=7,wi=15)

pearplot_befor1

pearplot_after1

qc_merge<- CombinePlots(plots = list(pearplot_befor1,pearplot_after1) ,

nrow=2, legend='none')

qc_merge

ggsave(filename = 'qc_merge.pdf',plot = qc_merge,he=9,wi=15)

save(datalist,file = 'datalist.RData')

sce <- merge(datalist[[1]],y=datalist[2:length(datalist)])

rm(datalist)

sce <- NormalizeData(sce, normalization.method = "LogNormalize", scale.factor = 10000)

sce <- FindVariableFeatures(sce,

selection.method = "vst",

nfeatures = 2000,

mean.cutoff=c(0.0125,3),

dispersion.cutoff =c(1.5,Inf))

top20 <- head(VariableFeatures(sce), 20)

plot1 <- VariableFeaturePlot(sce)

plot2 <- LabelPoints(plot = plot1, points = top20, repel = TRUE, size=3.0)

feat_20 <- CombinePlots(plots = list(plot1, plot2),legend="bottom")

feat_20

ggsave(filename = 'feat_20.pdf',plot = feat_20,he=10,wi=15)

#ScaleData

scale.genes <- rownames(sce)

sce <- ScaleData(sce, features = scale.genes)

meta1<-data.frame(matrix(nrow=length(sce@meta.data$orig.ident), ncol=2))

colnames(meta1)=c('Sample','Group1')

meta1$Sample=sce@meta.data$orig.ident

unique(meta1$Sample)

meta1[grep("N1",meta1$Sample),]$Group1="Normal"

meta1[grep("N2",meta1$Sample),]$Group1="Normal"

meta1[grep("T1",meta1$Sample),]$Group1="Tumor"

meta1[grep("T2",meta1$Sample),]$Group1="Tumor"

sce <- AddMetaData(sce, meta1$Sample,col.name = "Sample")

sce <- AddMetaData(sce, meta1$Group1,col.name = "Group1")

save(sce,file = 'sce.RData')

library(Seurat)

library(dplyr)

library(ggplot2)

library(magrittr)

library(gtools)

library(stringr)

library(Matrix)

library(tidyverse)

library(patchwork)

library(data.table)

library(RColorBrewer)

library(ggpubr)

load('sce.RData')

sce <- RunPCA(sce, features = VariableFeatures(sce))

dimplot1 <- DimPlot(sce, reduction = "pca")

elbowplot1 <- ElbowPlot(sce, ndims=50, reduction="pca")

sc_pca <- dimplot1+elbowplot1

sc_pca

ggsave(filename = 'sc_pca.pdf',plot = sc_pca,he=10,wi=15)

VizDimLoadings(sce, dims = 1:2, nfeatures = 20, reduction = "pca")

DimHeatmap(sce, dims = 1:20, cells = 500, balanced = TRUE)

Dims <- 40

Resolution <- 0.3

sce <- FindNeighbors(object = sce, dims = 1:Dims)

sce <- FindClusters(object = sce, resolution = Resolution)

allcolour=c("#DC143C","#0000FF","#20B2AA","#FFA500","#9370DB","#98FB98","#F08080","#1E90FF","#7CFC00","#FFFF00",

"#808000","#FF00FF","#CCCCFF","#000000","#7B68EE","#9400D3","#A0522D","#800080","#D2B48C","#D2691E",

"#87CEEB","#40E0D0","#5F9EA0","#FF1493","#0000CD","#008B8B","#FFE4B5","#8A2BE2","#228B22","#E9967A",

"#4682B4","#32CD32","#F0E68C","#FFFFE0","#EE82EE","#FF6347","#6A5ACD","#9932CC","#8B008B","#8B4513",

"#DEB887")

length(table(sce@active.ident))

mycolor = allcolour[1:length(table(sce@active.ident))]

cluster.frequency.table <- sce@meta.data %>%

dplyr::count(seurat_clusters) %>%

dplyr::mutate(freq = n / sum(n)*100) %>%

ungroup()%>%as.data.frame()

cluster.frequency.table

pie(cluster.frequency.table$n, labels=round(cluster.frequency.table$freq,2),radius=1.0, main = "Percentage of Cluster", col=mycolor)

legend("right",legend=unique(cluster.frequency.table$seurat_clusters),bty="n",fill=mycolor)

cluster.frequency.sample=data.frame()

for (i in as.character(unique(sce@meta.data$Group1))){

data1<-sce@meta.data[which(sce@meta.data$Group1==i),]

dat1 <- data1 %>%

dplyr::group_by(Group1) %>%

dplyr::count(seurat_clusters) %>%

dplyr::mutate(freq = n / sum(n)*100) %>%

ungroup()%>%as.data.frame()

cluster.frequency.sample=rbind(cluster.frequency.sample,dat1)

}

head(cluster.frequency.sample)

cluster.freq.sample<-tidyr::spread(data=cluster.frequency.sample[,c("Group1","seurat_clusters","freq")],

key=Group1, value=freq)

cluster.freq.sample[is.na(cluster.freq.sample)]<-0

head(cluster.freq.sample)

#rownames(cluster.freq.sample)=cluster.freq.sample$seurat_clusters

cluster.freq<-ggplot(data=cluster.frequency.sample, mapping=aes(x=Group1,y=freq,fill=seurat_clusters))+

geom_bar(stat='identity',width=0.9)+coord_polar(theta="y",start = 0)+

theme_bw() +

theme(panel.border = element_blank(),

panel.grid.major = element_blank(),

panel.grid.minor = element_blank(),

axis.line = element_blank())+

scale_fill_manual(values=mycolor)

cluster.freq

pdf('cluster_freq.pdf',he=7,wi=9)

cluster.freq

dev.off()

write.csv(cluster.frequency.sample,file ="cluster.frequency.csv")

### UMAP

sce <- RunUMAP(sce, dims=1:Dims, reduction="pca")

sce <- RunTSNE(sce,

dims=1:Dims,

reduction="pca",

perplexity=30,

max_iter=1000)

sc_umap = DimPlot(sce,cols=mycolor,

reduction="umap",

#reduction="tsne",

label = "T",

pt.size = 0.2,

label.size = 5) +

theme(axis.line = element_line(size=0.1, colour = "black"),

axis.ticks = element_blank()

)

sc_umap

ggsave('sc_umap_cluster.pdf',sc_umap,he=7,wi=7)

sc_tsne = DimPlot(sce,cols=mycolor,

#reduction="umap",

reduction="tsne",

label = "T",

pt.size = 0.2,

label.size = 5) +

theme(axis.line = element_line(size=0.1, colour = "black"),

axis.ticks = element_blank()

)

sc_tsne

ggsave('sc_tsne_cluster.pdf',sc_umap,he=7,wi=7)

sc_umap_group1 = DimPlot(sce,cols=mycolor,group.by='Sample',

reduction="umap",

label = "T",

pt.size = 0.2,

label.size = 0) +

theme(axis.line = element_line(size=0.1, colour = "black"),

axis.ticks = element_blank()

)

sc_umap_group1

ggsave('sc_umap_sample.pdf',sc_umap_group1,he=7,wi=7)

sc_umap_group2 = DimPlot(sce,cols=mycolor,group.by='Group1',

reduction="umap",

label = "T",

pt.size = 0.2,

label.size = 0) +

theme(axis.line = element_line(size=0.1, colour = "black"),

axis.ticks = element_blank()

)

sc_umap_group2

ggsave('sc_umap_group.pdf',sc_umap_group2,he=7,wi=7)

DefaultAssay(sce) <- "RNA"

sce.markers <- FindAllMarkers(object = sce,logfc.threshold = Logfc,

min.pct = Minpct,only.pos = T)

sce.markers["pct.diff"]=sce.markers$pct.1-sce.markers$pct.2

sce.markers <- sce.markers[sce.markers$p_val_adj<0.05,]

length(unique(sce.markers$gene))

head(sce.markers)

write.table(sce.markers,'scRNA_marker_gene.txt',quote = F,row.names = F,sep='\t')

Top5 <- sce.markers %>%

group_by(cluster) %>%

slice_max(n =5, order_by = avg_log2FC)

Top5 <- unique(Top5$gene)

sc_marker_dotplot <- DotPlot(object = sce,

features = Top5,

cols=c("blue", "red"),

scale = T)+

RotatedAxis()+ ggtitle("Top 5 Marker Genes")+

theme(plot.title = element_text(hjust = 0.5))

sc_marker_dotplot

ggsave(filename = 'sc_marker_dotplot.pdf',

plot = sc_marker_dotplot,

height = 9,width = 25)

library(viridisLite)

sc_marker_heatmap<- DoHeatmap(object = sce,

features = Top5,

group.colors = mycolor,

label = F) +

ggtitle("Top 5 Marker Genes") +

theme(plot.title = element_text(hjust = 0.5))

sc_marker_heatmap

ggsave(filename = 'sc_marker_heatmap.pdf',

plot = sc_marker_heatmap,

width = 12,height = 12)

save(sce,file = 'sce2.RData')
