# Supplementary material for: A novel risk score model based on fourteen chromatin regulators-based genes for predicting overall survival of patients with lower-grade gliomas
Source: Front Genet. 2022 Sep 26;13:957059. doi: 10.3389/fgene.2022.957059 (PMC9554745; doi:10.3389/fgene.2022.957059)
Supplement: Supplementary file 9 [file Table2.DOCX]

**Supplemental Table 2** The ten small molecule drugs extracted from the DSigDB database.

| **Term** | **P-value** | **Odds Ratio** | **Total score** | **N*** |
| --- | --- | --- | --- | --- |
| cephaeline HL60 DOWN | 1.49E-04 | 19.24 | 169.50 | 4 |
| emetine HL60 DOWN | 1.86E-04 | 18.15 | 155.91 | 4 |
| cephaeline MCF7 DOWN | 3.35E-04 | 27.26 | 218.05 | 3 |
| piroxicam CTD 00006571 | 4.51E-04 | 14.27 | 109.91 | 4 |
| emetine MCF7 DOWN | 5.91E-04 | 22.34 | 166.10 | 3 |
| formaldehyde CTD 00006001 | 0.003176 | 5.28 | 30.34 | 7 |
| NSC95682 | 0.003517 | 26.27 | 148.43 | 2 |
| phenobarbital CTD 00006510 | 0.006408 | 6.71 | 33.87 | 4 |
| hydrogen peroxide CTD 00006118 | 0.006513 | 4.87 | 24.52 | 6 |
| piperlongumine HL60 UP | 0.007123 | 18.14 | 89.67 | 2 |

P-value*: The error rates of prediction performance of drug–gene binding affinity prediction. Odds Ratio*: Odds ratio of prediction reliability. Total score*: Overall performance of drug prediction results. N*: Number of signature genes targeting the single-drug.
